# Genetic association of lipid-lowering drug target genes with erectile dysfunction and male reproductive health

**DOI:** 10.3389/fendo.2024.1362499

**Published:** 2024-02-08

**Authors:** Quanxin Su, Rui Wang, Yayin Luo, Qizhen Tang, Kenan Wang

**Affiliations:** ^1^ Department of Urology, The First Affiliated Hospital of Dalian Medical University, Dalian, Liaoning, China; ^2^ Department of Neurology, The First Affiliated Hospital of Dalian Medical University, Dalian, Liaoning, China

**Keywords:** erectile dysfunction, lipids, sex hormone, male diseases, Mendelian randomization analysis

## Abstract

**Objective:**

The effect of hypolipidemic drugs on male erectile function is still controversial. This Mendelian randomization (MR) study aimed to explore the potential impact of lipid-lowering drug targets on ED.

**Methods:**

We collected seven genetic variants encoding lipid-lowering drug targets (LDLR, HMGCR, NPC1L1, PCSK9, APOB, APOC3 and LPL) from published genome-wide association study (GWAS) statistics, and performed drug target MR analysis. The risk of ED was defined as the primary outcome, sex hormone levels and other diseases as the secondary outcomes. Mediation analyses were performed to explore potential mediating factors.

**Results:**

The results showed that LDLR, LPL agonists and APOC3 inhibitors were significantly associated with a reduced risk of ED occurrence. APOB inhibitors were associated with an increased risk of ED occurrence. In terms of sex hormone levels, LDLR and LPL agonists were significantly associated with increased TT levels, and HMGCR was associated with decreased TT and BT levels significantly. In terms of male-related disease, MR results showed that LDLR agonists and PCSK9 inhibitors were significantly associated with an elevated risk of PH; HMGCR, NPC1L1 inhibitors were associated with a reduced risk of PCa; and LDLR agonists were significantly associated with a reduced risk of AS and MI; in addition, HMGCR inhibitors were associated with a reduced risk of PCa.

**Conclusion:**

After performing drug-targeted MR analysis, we found that that there was a causal relationship between lipid-lowering drug targets and ED. APOC3, APOB, LDLR and LPL may be new candidate drug targets for the treatment of ED.

## Introduction

Erectile dysfunction (ED), defined as the persistent inability of the penis to achieve and/or maintain an erection sufficient for a satisfactory sex life, is one of the most common diseases in urology (1). Although ED does not pose a threat to life, it poses a significant safety hazard to society. It not only affects the physical and mental health of patients, but also causes great distress to sexual partners, leading to a decrease in the quality of life for patients and their partners, disharmony in the family, and more seriously, a decrease in work productivity, an increase in domestic violence, and an increase in medical burden. ED is highly related to cardiovascular risk factors such as hyperlipidemia, diabetes and abnormal blood pressure. Previous studies have found that the pathogenesis of ED and cardiovascular disease is basically the same, both centered on vascular endothelial dysfunction, ultimately leading to vascular atherosclerosis (2–4). Therefore, ED and cardiovascular disease share common risk factors. Lipids, including total cholesterol (TC), triglycerides (TG), low-density lipoprotein (LDL), and high-density lipoprotein (HDL), play a crucial role in this process.

Nicotinic acid, statins, fibrates and novel Lipid Lowering drugs are commonly used in the treatment of hyperlipidemia (5–8). There is clinical evidence that lipid-lowering drug therapy can significantly improve erectile function in patients with organic ED caused by hyperlipidemia (9, 10). However, some scholars have found that patients with hyperlipidemia may experience a decrease in testosterone levels during the use of lipid-lowering drugs, which in turn can lead to the occurrence of ED. Several meta-analyses have also shown similar conclusions (11, 12). In addition, some studies suggest that statins may indirectly lead to the occurrence of ED by affecting autonomic nervous function or psychological factors (13). Randomized controlled trials (RCTs) are the standard methods for determining drug efficacy and adverse reactions. However, there is currently a lack of large-scale randomized controlled trials between lipid-lowering drugs and ED. The impact of lipid-lowering drugs on the occurrence of ED and sex hormone levels is still unclear, and further exploration is needed.

With the increasing popularity of genome-wide association studies (GWAS), Mendelian randomization (MR) may be an effective alternative to RCT studies for problem solving. Because genetic variants (alleles) are randomly assigned during meiosis, participants in MR studies are “randomized” based on the presence of alleles. This is similar to a randomized controlled trial, where participants are randomly assigned to either an experimental treatment group or a control group (14, 15). Thus, MR analysis has the advantage of being less susceptible to confounding factors than other research methods. In recent years, drug target MR analysis has emerged as an effective tool. It is used to infer the effect of drugs targeting protein-coding genes, antagonists, agonists, or inhibitors on disease risk (16). This tool is very helpful in deciphering the potential of drug therapy and facilitating drug development.

In this study, we performed drug-targeted MR analysis to determine the effect of lipid-lowering drugs on ED and to explore the potential impact of lipid-lowering drug targets on sex hormone levels and other common male disorders.

## Materials and methods

### Study design

To explore the relationship between lipid-lowering drug target genes and male reproductive health at the genetic level, a two-sample Mendelian randomization approach was used in this study.

### Genetic variant selection

Based on the latest guidelines for the treatment of dyslipidemia, we selected common lipid-lowering drugs and novel therapeutic approaches such as statins, ezetimibe, PCSK9 inhibitors, mipomersen and antisense oligonucleotides targeting apolipoprotein C-III (APOC3) mRNA. The genes encoding the pharmacological targets of these drugs were identified using the DrugBank database. These target genes were further classified into LDL-c reducing target genes (i.e.; LDLR, HMGCR, NPC1L1, PCSK9 and APOB) and TG reducing target genes (i.e., LPL and APOC3) based on the primary pharmacological effect (**Table 1**).

**Table 1:** Basic information on lipid-lowering drugs.

Drug name	Pharmacological action	Drug targets	Target genes	Chromosome
—	Reduce LDL-C	LDL Receptor	LDLR	chr19:11,200,038-11,244,492
Simvastatin, Atorvastatin, Rosuvastatin	Reduce LDL-C	HMG-CoA reductase	HMGCR	chr5:74,632,154-74,657,929
Ezetimibe	Reduce LDL-C	Niemann-Pick C1-like protein 1	NPC1L1	chr7:44,552,134-44,580,914
Alirocumab, Evolocumab	Reduce LDL-C	Proprotein convertase subtilisin/kexin type 9	PCSK9	chr1:55,505,221-55,530,525
Mipomersen	Reduce LDL-C	Apolipoprotein B-100	APOB	chr2:21,224,301-21,266,945
—	Reduce TG	Lipoprotein Lipase	LPL	chr8:19,759,228-19,824,769
Volanesorsen	Reduce TG	Apolipoprotein C-III	APOC3	chr11:116,700,422-116,703,788

LDLR, low density lipoprotein receptor; HMGCR, HMG-CoA reductase; NPC1L1, Niemann-Pick C1-Like 1; PCSK9, proprotein convertase subtilisin-kexin type 9; APOB, Apolipoprotein (apo) B; LPL, Lipoprotein lipase; APOC3, antisense oligonucleotides targeting apolipoprotein C-III.

The summary data of LDL-C and TG were from two GWAS summary statistics containing 440,546 and 441,016 European individuals respectively (17). By obtaining instrumental variables that target each drug target to lower LDL-C or TC, they can be used to model the effects of each lipid-lowering drug. The instrumental variables were selected to be single nucleotide polymorphisms (SNP) located within ±100kb of the drug target locus and associated with LDL-C or TG levels (P< 10×5^−8^). To avoid the influence of strong linkage disequilibrium (LD) on the results, a threshold for LD was set (r^2^ < 0.3).

### Outcome

We used coronary heart disease (CHD) and ED as the results of drug-targeted MR analyses, where CHD was the positive control dataset to validate the feasibility and efficacy of lipid-lowering drug targets. The CHD dataset was derived from a GWAS summary statistic with a total of 184,305 cases, which contained 60,801 cases and 123,504 controls (18). ED as the main outcome, the data were from two independent GWAS datasets, respectively from FinnGen and ebi databases (19). Total testosterone (TT), bioavailable testosterone (BT), estrogen (E2) and SHBG were used as secondary outcomes, and their gender specific genetic instruments were from previously published UKB studies (20). Prostatitis (PI), prostate hyperplasia (PH), prostate cancer (PCa), aberrant spermatogenesis (AS), and male infertility (MI) equally performed as secondary outcomes with genetic instrumentation from previously published studies and the FinnGen database (21). Detailed information can be found in **Supplementary Table 1**.

### Estimation of causal effects

We used the inverse variance weighted method (IVW) to estimate the causal effect between the drug targets and ED. In addition, we performed additional analyses by weighted median method and weighted model (22–24). Sufficient evidence of a causal effect was consistently provided by statistically significant IVW results plus direction of results across all 3 analyses. We also conducted Steiger filtering to ensure directional correlation between medication and outcomes.

### Meta-analysis

The ‘metafor’ package was used to analyze the data indicators, and the Risk ratio OR was used as the merging statistic (25). When I^2^>50%, the study was considered to have greater heterogeneity, and the results of the combined analysis were analyzed using the random effects model for Meta-analysis, and when I^2^<50%, the study was considered to have homogeneity, and the results of the combined analysis were analyzed using the fixed effects model.

### Quality controls

Heterogeneity was tested using MR Egger and IVW methods. Cochrane’s Q was used to evaluate the heterogeneity of the genetic tools, and p>0.05 indicated the absence of significant heterogeneity. MR Egger regression equation was used to evaluate the horizontal multiplicity of genetic tools and p > 0.05 indicated the absence of horizontal multiplicity. To ensure that our results were not significantly affected by a particular SNP, we also removed each SNP in turn using the leave-one-out method and compared the results of the IVW method with all variants.

### Mediation MR analysis

**Figure 1:**
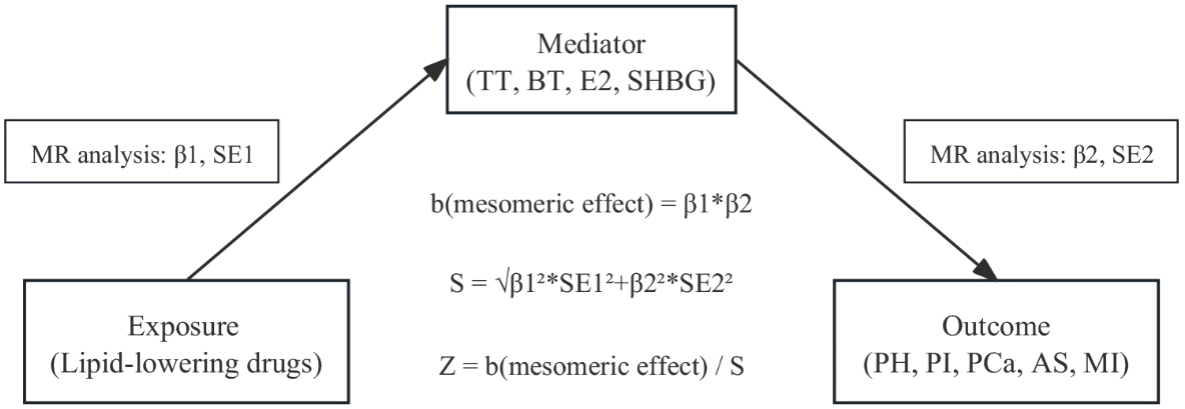
Mediation analysis of the effect of drug targets on male diseases.

To determine whether there was a direct relationship between the observed associations between drugs and outcomes, we used a “two-sample” approach to assess potential intermediate effects (exposure-mediated-outcome pathways) of drugs on established outcome variables (**Figure 1**). This method reduces the bias of high LD correlations between genetic variants in MR analyses compared with multivariate MR methods. The method uses a “product of coefficients” approach to evaluate indirect effects and a delta method to derive standard errors for indirect effects (26).

The statistical analysis in this study was conducted using RStudio software (version 4.1.2). The resources used were mainly obtained from the TwoSampleMR R package developed by Hemani et al.

## Results

### Positive control analysis

**Figure 2:**
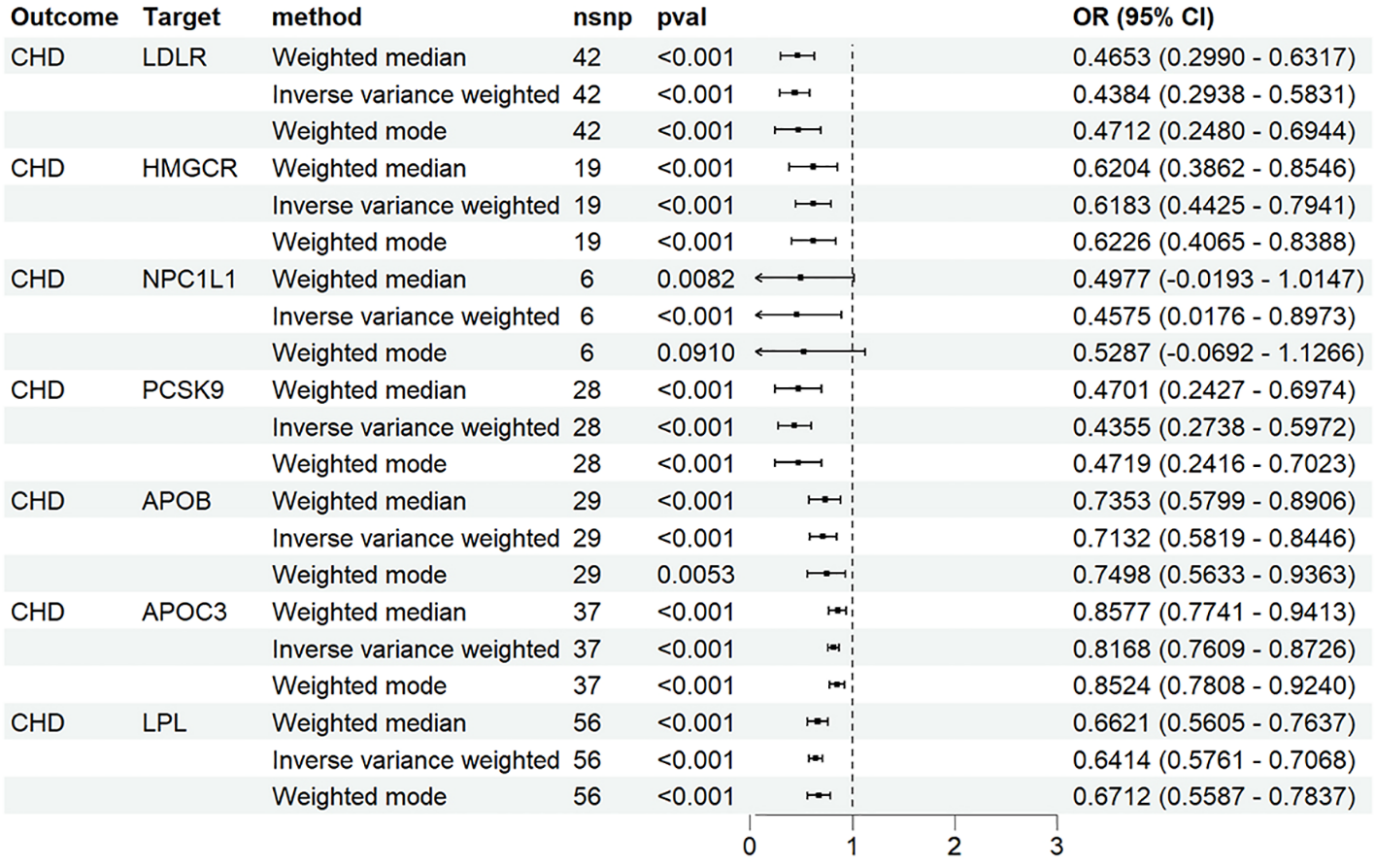
MR analysis of association between drug targets and CHD.

We identified 48 SNPs associated with LDLR agonists, 19 SNPs associated with HMGCR inhibitors, 6 SNPs associated with NPC1L1 inhibitors, 33 SNPs associated with PCSK9 inhibitors, and 32 SNPs associated with apoB inhibitors from the genetic tools of LDL-C; Forty SNPs associated with APOC3 inhibitors and 58 SNPs associated with LPL agonists were identified from the genetic tools of TG. In the MR analysis with CHD as the outcome, as expected, the results of IVW showed that the seven drugs significantly reduced the risk of CHD (**Figure 2**).

### The causal relationship between lipid-lowering drug targets and primary outcomes

According to the IVW results of the primary analysis method, LDLR agonists showed a significant association with a reduced risk of ED occurrence in the ebi database (OR [95%] = 0.755 [0.559-0.951], p=0.005). In the FinnGen database, the APOC3 inhibitors reduced the risk of ED occurrence (OR [95%] = 0.733 [0.439-1.026], p=0.038). In contrast, APOB inhibitors showed a significant association with an elevated risk of ED occurrence (OR [95%] = 2.77 [2.289-3.251], p<0.001). Results of MR analysis are shown in **Table 2**.

**Table 2:** The effect of drug targets on ED.

Drug target	Methods	ED (FinnGen database)		ED (ebi database)	
		OR (95% CI)	P value	OR (95% CI)	P value
LDLR	Inverse variance weighted	0.834 (0.375, 1.294)	0.44	0.755 (0.559, 0.951)	0.005
	Weighted median	1.109 (0.426, 1.791)	0.767	0.852 (0.563, 1.14)	0.275
	Weighted mode	1.222 (0.532, 1.913)	0.572	0.896 (0.562, 1.23)	0.523
HMGCR	Inverse variance weighted	1.768 (1.032, 2.504)	0.129	0.716 (0.364, 1.068)	0.063
	Weighted median	1.63 (0.653, 2.608)	0.327	0.821 (0.339, 1.304)	0.425
	Weighted mode	1.715 (0.73, 2.7)	0.297	0.762 (0.282, 1.242)	0.282
NPC1L1	Inverse variance weighted	2.12 (0.412, 3.827)	0.389	1.191 (0.346, 2.037)	0.685
	Weighted median	1.544 (-0.481, 3.569)	0.674	0.911 (-0.208, 2.03)	0.871
	Weighted mode	1.327 (-0.845, 3.5)	0.808	0.882 (-0.406, 2.169)	0.855
PCSK9	Inverse variance weighted	0.933 (0.494, 1.373)	0.759	1.222 (0.935, 1.509)	0.170
	Weighted median	0.818 (0.24, 1.396)	0.495	1.466 (1.069, 1.863)	0.059
	Weighted mode	0.836 (0.291, 1.381)	0.524	1.481 (1.108, 1.853)	0.048
APOB	Inverse variance weighted	2.77 (2.289, 3.251)	<0.001	1.034 (0.752, 1.316)	0.816
	Weighted median	3.097 (2.529, 3.665)	<0.001	0.818 (0.438, 1.197)	0.298
	Weighted mode	3.182 (2.562, 3.803)	0.001	0.796 (0.385, 1.206)	0.289
LPL	Inverse variance weighted	0.932 (0.641, 1.223)	0.635	0.906 (0.775, 1.037)	0.138
	Weighted median	1.152 (0.729, 1.576)	0.511	0.96 (0.775, 1.146)	0.67
	Weighted mode	1.092 (0.676, 1.509)	0.679	0.958 (0.775, 1.141)	0.647
APOC3	Inverse variance weighted	0.733 (0.439, 1.026)	0.038	0.894 (0.767, 1.022)	0.087
	Weighted median	0.804 (0.410, 1.197)	0.276	0.92 (0.745, 1.095)	0.352
	Weighted mode	0.801 (0.393, 1.210)	0.296	0.909 (0.743, 1.075)	0.267

LDLR, low density lipoprotein receptor; HMGCR, HMG-CoA reductase; NPC1L1, Niemann-Pick C1-Like 1; PCSK9, proprotein convertase subtilisin-kexin type 9; APOB, Apolipoprotein (apo) B; LPL, Lipoprotein lipase; APOC3, antisense oligonucleotides targeting apolipoprotein C-III.

**Figure 3:**
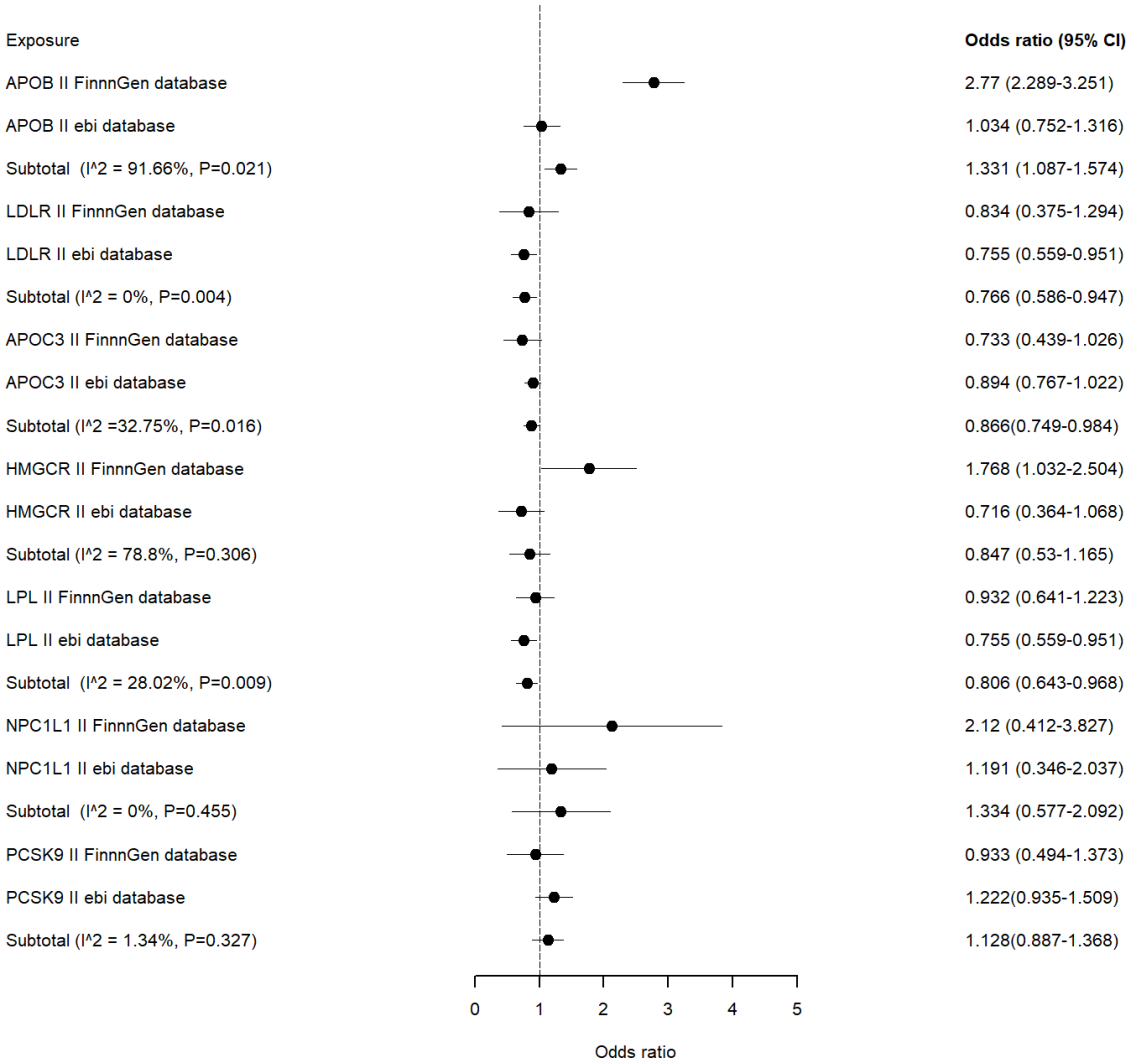
Meta analysis of association between drug targets and ED.

Meta-analysis was performed on the MR results obtained from each of the two databases. The combined analysis showed that LDLR, LPL agonists and APOC3 inhibitors were significantly associated with a reduced risk of ED occurrence (OR [95%] = 0.766 [0.586 - 0.947], p=0.004 vs OR [95%] = 0.806 [0.643-0.968], p=0.009 vs OR [95%] = 0.866 [0.749-0.984], p=0.016) (**Figure 3**).APOB inhibitors were significantly associated with an increased risk of ED occurrence (OR[95%]=1.331 [1.087-1.574], p=0.021). Other inhibitors were not significantly associated with the risk of ED occurrence.

### The causal relationship between lipid-lowering drug targets and secondary outcomes

In the results of MR analysis with sex hormone levels as an outcome, LDLR and LPL agonists were significantly associated with increased TT levels (OR[95%] = 1.048 [1.019-1.078], p=0.001 vs OR[95%] = 2.77 [2.289-3.251], p<0.001), and HMGCR was significantly associated with decreased TT and BT levels significantly (OR[95%] = 1.08 [1.060-1.1006], p<0.001 vs OR[95%] = 0.824 [0.764-0.884], p<0.001).LDLR, LPL agonists, and PCSK9, APOC3 inhibitors were significantly correlated with elevated SHBG levels, while HMGCR was decreased significantly. In addition, no significant causal relationship was found between drugs and E2 levels (**Supplementary Figures 12**, **13**).

In studies with male-related disease as an outcome, MR results showed that LDLR agonists and PCSK9 inhibitors were significantly associated with an elevated risk of PH occurrence (OR [95%]=1.362 [1.196-1.527], p<0.001 vs OR [95%]=1.261 [1.095-1.427], p=0.006); HMGCR, NPC1L1 inhibitors were significantly associated with a reduced risk of developing PCa (OR [95%] = 0.616 [0.399-0.833], p<0.001 vs OR [95%] = 0.427 [-0.23-1.088], p=0.01); and LDLR agonists were significantly associated with a reduced risk of developing AS and MI (OR [95%] = 0.473 [-0.028 - 0.974], p=0.003 vs OR[95%] = 0.412 [-0.192 - 1.015], p=0.003); in addition, HMGCR inhibitors were significantly associated with a reduced risk of developing PCa (OR[95%] = 7.239 [6.286-8.193], p< 0.001) (**Supplementary Figures 14**, **15**).

### Sensitivity analysis

Cochrane’s Q and MR Egger regression equations were used to assess levels of heterogeneity and horizontal pleiotropy. When we examined the causal relationship between APOC3 inhibitors and ED (FennGen), we found significant horizontal pleiotropy (p=0.03) (**Supplementary Table 11**). When we examined the causal relationship between PCSK9, APOB inhibitors and ED(ebi), we found significant horizontal pleiotropy (p=0.03 vs p=0.03). Therefore, in order to obtain more reliable results, we used more stringent criteria for selecting instrumental variables, changing the LD parameters from r^2^<0.3 to r^2^<0.2 (PCSK9 and APOC3) and r^2^<0.1 (APOB). MR analysis was performed again, and the updated results did not show significant heterogeneity or horizontal pleiotropy, and the updated results will be used for subsequent Meta-analysis (**Supplementary Table 12**).

The results of the sensitivity analysis also showed no heterogeneity or horizontal pleiotropy (p>0.05) in all other outcomes (MR analysis between drugs and hormones, MR analysis between drugs and other male diseases) (**Supplementary Tables 14**, **16**).

The leave-one-out method showed that there would be no significant difference in the results after removing any SNP (**Supplementary Figures 1**–**11**).

### Mediation analysis

Given the close relationship between sex hormones and male erectile function, reproductive function, and some male disease interest, they may mediate the effect of lipid-lowering drugs on male disease risk. We therefore used the coefficient product method of mediation analysis to examine the mediating pathway from drug to disease. The results of the mediation analyses suggest that lipid-lowering drugs do not influence the risk of disease development by altering hormone levels. Sex hormone levels were not a mediator between lipid-lowering drugs and disease occurrence (**Supplementary Table 18**).

## Discussion

Erectile dysfunction is a common disease in urology and andrology, which not only brings great pain to the patient and his family, but also is not conducive to the stability of society and family. Therefore, the epidemiology and pathogenesis of ED have attracted more and more attention from scholars all over the world. A large number of experimental and clinical data show that hyperlipidemia is an important risk factor for erectile dysfunction. Current studies have shown that hyperlipidemia induced arterial stenosis and occlusion may only be the late mechanism of hyperlipidemia induced ED. Hyperlipidemia can affect the endothelial cells, smooth muscle cells and peripheral nerves of the penis in the early stage and damage the erectile function of the penis (27–29).

Firstly this study identified two potential targets, low density lipoprotein receptor (LDLR) and Lipoprotein lipase (LPL), which are significantly associated with the development of ED by drug target MR analysis. LPL is a key enzyme regulating lipid fuel processing, and decreased levels or activity can alter LPL lipolytic function, leading to hyperlipidemia and metabolic disorders in the body, causing damage to the vascular endothelium (30). Lorentzen et al.’s study concluded that high blood lipids can increase endothelial cell activity or sensitivity (31). *In vitro* experiments also demonstrated that high plasma TG can damage vascular endothelial cells (32). Therefore, we hypothesized that LPL gene agonists could increase LPL enzyme activity, reduce plasma TG levels, and protect vascular endothelial function, thereby achieving a reduction in the risk of ED. Similar to LPL, LDLR is a key receptor for the body to remove LDL-C from plasma by endocytosis, and it is one of the 3 genes known to be associated with autosomal dominant inheritance of familial hypercholesterolemia (33, 34). Increased LDLR expression reduces plasma cholesterol levels. Musicki et al. found that LDLR-deficient mice fed a high cholesterol diet had a significantly reduced erectile response, and further studies found significantly increased levels of oxidative stress in the penises of mice (35). Similar results were obtained in the present study that LDLR agonists significantly reduced the risk of CHD development and were significantly associated with a reduced risk of ED development.

3-Hydroxy-3-methylglutaryl coenzyme A reductase (HMGCR) inhibitors, also known as statins, are the first-line drugs used for the prevention and treatment of cardiovascular diseases (36, 37). Currently, the main statins used in clinical practice are lovastatin, simvastatin, pravastatin, fluvastatin and atorvastatin. As early as 1996, Bruckert et al. reported the first case of statin-induced ED, and subsequently divided 678 hyperlipidemic male patients aged 30-70 years into a lipid-lowering drug group and a placebo group (339 cases in each group), and found that the incidence of ED in the lipid-lowering drug group was 12.1%, and the placebo group was 5.6%, with the difference statistically significant (P=0.0029) (38). Rizvi et al. have also reported 5 patients with ED triggered by simvastatin (10 mg/d and 20 mg/d), which returned to normal after 1 week of discontinuation of the drug (2 of them had ED recurrence in the provocation test) (11). However, in the present study, MR analysis did not reveal a significant causal relationship between HMGCR inhibitors and ED occurrence. We believe that most of the patients reported in the previous literature had ED risk factors such as hyperlipidemia and essential hypertension, and the increased incidence of ED may be a result of the combined effect of the above multifactorial factors, which should not be simply attributed to lipid-lowering drugs alone. And simvastatin listed earlier, can not completely represent other statin drugs. Some scholars believe that statins cause ED may be related to their inhibition of HMG-CoA reductase to reduce cholesterol synthesis, affecting the synthesis of steroid hormones (including testosterone).Corona et al. found that 244 cases of statin users serum total testosterone and free testosterone concentrations were significantly lower than normal values (39). In the present study, MR analysis showed that HMGCR inhibitors were associated with significantly lower levels of TT, BT, and SHBG, consistent with previous studies. In addition, the non-lipid-lowering effects of statins (e.g., antioxidant effects) have received increasing attention. The results of foreign studies in recent years have shown that statins can reduce the risk of prostate cancer, decrease the recurrence rate of prostate cancer, and delay the progression of the disease (40, 41). The results of this study are consistent with previous findings, but the specific mechanism of action is not clear.

Currently lipid-lowering drugs still recommend statins as the first-line therapeutic drugs, but there are some patients who are intolerant to statins or have poor therapeutic effects (8). With the development of new drugs, some non-statin lipid-lowering drugs have been gradually applied to clinical practice. Since the identification of the causative gene, proprotein convertase subtilisin-kexin type 9 (PCSK9), in familial hypercholesterolemia family lines in 2003, the development of PCSK9-targeted inhibitors has progressed rapidly (42, 43). PCSK9 inhibitors are now widely used as a new class of lipid-lowering drugs in a wide range of patients with cardiovascular diseases, including familial hypercholesterolemia. A study by Mostaza et al. found that the R46L variant of PCSK9 was significantly associated with the development of ED, and that the prevalence of ED was higher in carriers of the T allele of the R46L gene (44). And in a real-world study, Scicali et al. evaluated the effect of PCSK9-i on sexual function as assessed by the Men’s Sexual Health Questionnaire (MSHQ) and the International Index of Erectile Function (IIEF-5) questionnaires, and found that patients’ MSHQs improved with the addition of PCSK9-i treatment, whereas there was no significant difference in the IIEF-5 before and after use (45). In the present study, MR analysis similarly did not find a significant causal relationship between PCSK9-i and the development of ED. In addition the present study also found that PCSK9-i and SHBG levels were associated with an elevated risk of PH development, but the exact mechanism still needs to be explored subsequently.

ApoC III is a strong inhibitor of LPL, which hinders the breakdown and clearance of TRLs and leads to the aggregation of circulating pro-atherosclerotic factors such as coeliac particles and VLDL. Numerous studies at home and abroad have shown that elevated serum ApoC III levels can cause hypertriacylglycerolemia, and that inhibiting ApoC III synthesis or lowering blood (46–48). Levels of ApoC III is an effective strategy for the prevention of cardiovascular disease. In the present study, ApoC III inhibitors were found to significantly reduce the risk of ED. APOB also plays a central role in human lipoprotein metabolism. The APOB gene produces two forms of apoB through a unique post-transcriptional editing process: apoB-48 and apoB-100.ApoB-100 is an important structural component of very low-density lipoproteins (VLBIs) and their metabolites, intermediate-density lipoproteins (IDL) and ApoB-100 is an important structural component of LDL and its metabolites, intermediate density lipoprotein (IDL) and LDL, as well as a ligand for receptor-mediated endocytosis of LDL (49). Increased plasma concentrations of ApoB have been shown to be a key risk factor for the development of atherosclerosis. Mipomersen, an antisense oligodeoxynucleotide inhibitor of apolipoprotein B-100, has been approved for the treatment of familial hypercholesterolemia (50). Some studies have suggested a significant negative correlation between ApoB and testosterone levels (51, 52), but the present study did not find a significant causal relationship between ApoB inhibitors and testosterone levels. It is noteworthy that ApoB inhibitors were the only lipid-lowering agents found to be associated with an increased risk of ED development in the MR analysis results of this study, and this result and the specific mechanism still need to be verified in subsequent clinical trials.

Our study has some unavoidable limitations. First, MR analysis is only a method for analyzing causal relationships between exposures and outcomes, and it is more useful for determining the direction of the association than for quantifying the magnitude of the association. It cannot completely replace clinical trials in the objective world. Secondly, drug-targeted MR analysis may not accurately reflect the effects of short-term and different routes of administration. Finally, due to insufficient GWAS data resources, we only performed MR analysis on European populations and our findings may not be applicable to other ethnicities.

## Conclusion

In conclusion, after performing drug-targeted MR analysis, we found that APOC3 inhibitors among lipid-lowering drugs significantly reduced the risk of ED occurrence, while on the contrary APOB inhibitors may be associated with an elevated risk of ED occurrence. In addition, the results also found that PCSK9 inhibitors increased the risk of PH, and NPC1L1, HMGCR inhibitors decreased the risk of PCa, but at the same time HMGCR inhibitors increased the risk of male infertility. Notably, this study suggests that LDLR and LPL may be new candidate drug targets for the treatment of ED, and their agonists significantly reduced the risk of ED occurrence in MR analysis, but the results still need to be further validated in basic, clinical studies.

## Data availability statement

The datasets presented in this study can be found in online repositories. The names of the repository/repositories and accession number(s) can be found in the article/**Supplementary Material**.

## Ethics statement

Ethical approval was not required for the study involving humans in accordance with the local legislation and institutional requirements. Written informed consent to participate in this study was not required from the participants or the participants’ legal guardians/next of kin in accordance with the national legislation and the institutional requirements.

## Author contributions

QS: Writing – original draft, Writing – review & editing. RW: Writing – review & editing. YL: Writing – review & editing. QT: Writing – original draft. KW: Writing – review & editing.
