# Supplementary material for: Genetic association of lipid-lowering drug target genes with erectile dysfunction and male reproductive health
Source: Front Endocrinol (Lausanne). 2024 Feb 8;15:1362499. doi: 10.3389/fendo.2024.1362499 (PMC10881712; doi:10.3389/fendo.2024.1362499)
Supplement: Supplementary file 1 [file Table_1.docx]

**Supplementary Table 1.** Summary of lipids, male diseases and sex hormones.

| Category | Variable | Population | Number of SNPs | Sample Size | Year | Consortium |
| --- | --- | --- | --- | --- | --- | --- |
| Lipids | LDL-C | European | 12,321,875 | 440,546 | 2020 | UK Biobank |
|  | Triglycerides | European | 12,321,875 | 441,016 | 2020 | UK Biobank |
| Male diseases | Erectile dysfunction | European | 16,378,833 | 95,178 | 2021 | FinnGen |
|  |  | European | 9,310,196 | 223,805 | 2018 | ebi |
|  | Prostatic hyperplasia | European | 16,378,414 | 85,917 | 2021 | FinnGen |
|  | Prostatitis | European | 16,377,460 | 74,658 | 2021 | FinnGen |
|  | Prostatic cancer | European | 24,119,306 | 211,227 | 2021 | ebi |
|  | Abnormal sperm | European | 16,380,442 | 209,921 | 2021 | FinnGen |
|  | Male infertility | European | 16,377,329 | 73,479 | 2021 | FinnGen |
| Sex hormone | Total Testosterone | European | 12,321,875 | 199,569 | 2020 | UKB |
|  | Bioavailable Testosterone | European | 12,321,875 | 184,205 | 2020 | UKB |
|  | Oestradiol | European | 12,321,875 | 17,134 | 2020 | UKB |
|  | Sex hormone binding globulin | European | 12,321,875 | 185,221 | 2020 | UKB |

**Supplementary Table 2.** The detail of instrumental variable corresponding to LDLR.

| chr.exposure | pos.exposure | beta.exposure | se.exposure | pval.exposure | SNP | effect_allele.exposure | other_allele.exposure | eaf.exposure |
| --- | --- | --- | --- | --- | --- | --- | --- | --- |
| 19 | 11164968 | -0.0134092 | 0.00245241 | 4.60002E-08 | rs2421198 | T | C | 0.232896 |
| 19 | 11174441 | -0.0236774 | 0.00419212 | 0.000000016 | rs73013196 | T | C | 0.067976 |
| 19 | 11190764 | 0.0427212 | 0.00402001 | 2.19989E-26 | rs36005514 | A | G | 0.071349 |
| 19 | 11232261 | 0.0644983 | 0.0109407 | 3.69999E-09 | rs147223423 | T | A | 0.009169 |
| 19 | 11284302 | -0.0189721 | 0.00206993 | 4.90004E-20 | rs379309 | T | C | 0.501714 |
| 19 | 11302606 | 0.016716 | 0.00208789 | 1.20005E-15 | rs7250652 | G | A | 0.443277 |
| 19 | 11146499 | -0.0305197 | 0.00208477 | 1.59993E-48 | rs3786721 | C | T | 0.557713 |
| 19 | 11147526 | -0.17308 | 0.00989319 | 1.59993E-68 | rs73013176 | C | T | 0.011456 |
| 19 | 11158047 | -0.0165393 | 0.00235425 | 2.09991E-12 | rs10420325 | T | A | 0.639284 |
| 19 | 11169305 | 0.0360512 | 0.00527004 | 7.89951E-12 | rs36049922 | C | T | 0.040127 |
| 19 | 11196394 | 0.068455 | 0.0108168 | 2.5E-10 | rs139760949 | A | C | 0.009752 |
| 19 | 11257018 | 0.0141311 | 0.0023743 | 2.69998E-09 | rs11557092 | C | T | 0.735703 |
| 19 | 11264618 | 0.0455464 | 0.00547214 | 8.60003E-17 | rs140181075 | T | C | 0.037619 |
| 19 | 11266693 | 0.0416562 | 0.00225634 | 4.20049E-76 | rs7251031 | G | T | 0.306748 |
| 19 | 11267940 | -0.0316003 | 0.0025504 | 2.90001E-35 | rs11673593 | T | A | 0.790703 |
| 19 | 11269893 | -0.0572931 | 0.00211261 | 5.7943E-162 | rs76213248 | T | C | 0.410251 |
| 19 | 11123091 | 0.0239223 | 0.0040324 | 2.99999E-09 | rs117819913 | A | T | 0.07124 |
| 19 | 11126160 | -0.0889437 | 0.00415804 | 1.5996E-101 | rs73013166 | C | T | 0.066524 |
| 19 | 11133827 | 0.0346014 | 0.00290269 | 9.30037E-33 | rs12609589 | T | C | 0.153388 |
| 19 | 11152888 | -0.0437918 | 0.00432384 | 4.19952E-24 | rs144624209 | A | G | 0.064106 |
| 19 | 11158055 | 0.0265587 | 0.00399608 | 2.99985E-11 | rs4804564 | T | C | 0.927533 |
| 19 | 11183837 | -0.0576403 | 0.00241122 | 2.6977E-126 | rs73015007 | A | G | 0.250898 |
| 19 | 11185919 | -0.103305 | 0.00270769 | 1E-200 | rs10423733 | C | T | 0.180142 |
| 19 | 11204627 | 0.0315779 | 0.00420565 | 6.00067E-14 | rs17242367 | T | C | 0.070321 |
| 19 | 11207516 | -0.112139 | 0.00553933 | 4.00037E-91 | rs73015030 | A | G | 0.036317 |
| 19 | 11227480 | 0.0422734 | 0.00211024 | 2.90001E-89 | rs2738447 | C | A | 0.592612 |
| 19 | 11230690 | 0.0681552 | 0.00524329 | 1.20005E-38 | rs116959285 | G | C | 0.045717 |
| 19 | 11235423 | 0.0239253 | 0.00216633 | 2.29985E-28 | rs6511724 | C | T | 0.642857 |
| 19 | 11277922 | 0.029107 | 0.00481642 | 1.5E-09 | rs111731690 | T | G | 0.05169 |
| 19 | 11284028 | 0.0153178 | 0.00229125 | 2.29985E-11 | rs4804149 | C | T | 0.287226 |
| 19 | 11335477 | -0.0450814 | 0.00559272 | 7.59976E-16 | rs8101801 | A | C | 0.035559 |
| 19 | 11116266 | 0.0192288 | 0.00331813 | 6.80002E-09 | rs13345127 | T | C | 0.109899 |
| 19 | 11127139 | -0.048528 | 0.00677934 | 8.19974E-13 | rs117234045 | A | G | 0.025203 |
| 19 | 11206040 | -0.0656844 | 0.00830137 | 2.49977E-15 | rs17248748 | T | C | 0.015825 |
| 19 | 11206575 | -0.0474014 | 0.00207343 | 1.099E-115 | rs6511721 | A | G | 0.519695 |
| 19 | 11241428 | 0.0531066 | 0.00378972 | 1.29987E-44 | rs17249001 | A | G | 0.088845 |
| 19 | 11242307 | 0.0257528 | 0.00317135 | 4.60045E-16 | rs2738464 | C | G | 0.87843 |
| 19 | 11248104 | -0.0710448 | 0.00631556 | 2.29985E-29 | rs147540853 | A | G | 0.027789 |
| 19 | 11257169 | -0.0704358 | 0.00264596 | 3.9995E-156 | rs79668907 | T | C | 0.228499 |
| 19 | 11175823 | -0.0965655 | 0.0126099 | 1.9002E-14 | rs56315738 | T | C | 0.006737 |
| 19 | 11176337 | 0.0451213 | 0.00470452 | 8.69961E-22 | rs11671812 | T | C | 0.051222 |
| 19 | 11184250 | -0.0486439 | 0.00210973 | 1.3002E-117 | rs2421201 | G | A | 0.501212 |
| 19 | 11192603 | -0.14759 | 0.00830891 | 1.39991E-70 | rs112159161 | T | C | 0.016621 |
| 19 | 11211077 | 0.0414739 | 0.00433606 | 1.10002E-21 | rs3745677 | A | G | 0.061743 |
| 19 | 11257499 | 0.0450148 | 0.00694567 | 9.09913E-11 | rs141618758 | C | T | 0.024108 |
| 19 | 11282298 | -0.060965 | 0.00817716 | 8.99912E-14 | rs117339792 | A | G | 0.01984 |
| 19 | 11283222 | -0.107272 | 0.00482702 | 1.9999E-109 | rs146576912 | T | C | 0.060983 |
| 19 | 11336626 | -0.0201813 | 0.00367256 | 3.89996E-08 | rs1433091 | G | A | 0.906073 |

**Supplementary Table 3.** The detail of instrumental variable corresponding to HMGCR.

| chr.exposure | pos.exposure | beta.exposure | se.exposure | pval.exposure | SNP | effect_allele.exposure | other_allele.exposure | eaf.exposure |
| --- | --- | --- | --- | --- | --- | --- | --- | --- |
| 5 | 74624484 | -0.0372115 | 0.00487202 | 2.19989E-14 | rs75240579 | T | C | 0.048363 |
| 5 | 74562029 | 0.03556 | 0.00261075 | 2.99985E-42 | rs2006760 | G | C | 0.205486 |
| 5 | 74664987 | -0.0271295 | 0.00433093 | 3.69999E-10 | rs62366588 | A | C | 0.065948 |
| 5 | 74615209 | 0.0532822 | 0.00653971 | 3.69999E-16 | rs141642272 | C | G | 0.026705 |
| 5 | 74651864 | 0.042154 | 0.0029315 | 6.89922E-47 | rs55727654 | A | G | 0.14871 |
| 5 | 74623949 | 0.0243909 | 0.00372718 | 6.00067E-11 | rs111353455 | A | G | 0.085844 |
| 5 | 74641707 | 0.0333589 | 0.00345272 | 4.40048E-22 | rs2303152 | A | G | 0.101442 |
| 5 | 74729433 | -0.0303618 | 0.00499242 | 1.2E-09 | rs116153450 | A | C | 0.04623 |
| 5 | 74656539 | 0.0621175 | 0.00212705 | 1.6982E-187 | rs12916 | C | T | 0.400537 |
| 5 | 74682474 | 0.0394972 | 0.00635898 | 5.30005E-10 | rs17562727 | C | T | 0.027617 |
| 5 | 74717761 | -0.0260509 | 0.00385694 | 1.39991E-11 | rs80324692 | T | C | 0.081157 |
| 5 | 74563700 | 0.048608 | 0.00785612 | 6.1E-10 | rs115845757 | A | G | 0.019 |
| 5 | 74650106 | 0.0619849 | 0.00619367 | 1.39991E-23 | rs17648121 | T | C | 0.029877 |
| 5 | 74682600 | 0.0329927 | 0.00582168 | 0.000000015 | rs140092661 | T | A | 0.034128 |
| 5 | 74757657 | 0.0251785 | 0.00459392 | 4.20001E-08 | rs12659331 | C | A | 0.054326 |
| 5 | 74630829 | 0.0564278 | 0.00316653 | 4.90004E-71 | rs72633963 | A | G | 0.1238 |
| 5 | 74560487 | 0.0410063 | 0.00216518 | 5.40008E-80 | rs10051965 | T | C | 0.369925 |
| 5 | 74610293 | -0.0281057 | 0.00423755 | 3.29989E-11 | rs35122945 | C | A | 0.067368 |
| 5 | 74602898 | 0.0244938 | 0.00297742 | 1.9002E-16 | rs4703665 | C | T | 0.848709 |

**Supplementary Table 4.** The detail of instrumental variable corresponding to NPC1L1.

| chr.exposure | pos.exposure | beta.exposure | se.exposure | pval.exposure | SNP | effect_allele.exposure | other_allele.exposure | eaf.exposure |
| --- | --- | --- | --- | --- | --- | --- | --- | --- |
| 7 | 44592091 | -0.0213142 | 0.00211012 | 5.50047E-24 | rs217399 | T | C | 0.443739 |
| 7 | 44596644 | 0.0259282 | 0.00392582 | 4.00037E-11 | rs73107478 | C | A | 0.079159 |
| 7 | 44570067 | -0.0133498 | 0.00234076 | 0.000000012 | rs11763759 | C | T | 0.303527 |
| 7 | 44582331 | 0.0355498 | 0.00267287 | 2.29985E-40 | rs2073547 | G | A | 0.184007 |
| 7 | 44559803 | 0.017365 | 0.00256025 | 1.20005E-11 | rs148825701 | T | C | 0.212133 |
| 7 | 44586578 | 0.0249436 | 0.00363876 | 7.10068E-12 | rs12666108 | C | T | 0.091516 |

**Supplementary Table 5.** The detail of instrumental variable corresponding to PCSK9.

| chr.exposure | pos.exposure | beta.exposure | se.exposure | pval.exposure | SNP | effect_allele.exposure | other_allele.exposure | eaf.exposure |
| --- | --- | --- | --- | --- | --- | --- | --- | --- |
| 1 | 55433978 | -0.0234719 | 0.00358388 | 5.79963E-11 | rs6691964 | A | G | 0.092472 |
| 1 | 55491135 | 0.0175746 | 0.00243048 | 4.79954E-13 | rs556369867 | T | C | 0.330585 |
| 1 | 55494301 | -0.0334061 | 0.00501479 | 2.70023E-11 | rs72909541 | T | C | 0.046097 |
| 1 | 55520938 | 0.0452728 | 0.00520209 | 3.19963E-18 | rs150119739 | A | G | 0.045318 |
| 1 | 55522558 | 0.0454642 | 0.0075822 | 0.000000002 | rs7525503 | T | G | 0.02036 |
| 1 | 55522674 | -0.0282322 | 0.00279415 | 5.30029E-24 | rs11587071 | T | C | 0.168881 |
| 1 | 55538552 | -0.0531381 | 0.00394676 | 2.60016E-41 | rs10493176 | G | T | 0.07579 |
| 1 | 55489960 | -0.0297494 | 0.00231882 | 1.10002E-37 | rs3976734 | G | A | 0.374504 |
| 1 | 55491853 | -0.0543492 | 0.00278155 | 5.10035E-85 | rs200730299 | C | A | 0.195034 |
| 1 | 55496131 | 0.0305717 | 0.00365832 | 6.4003E-17 | rs17192725 | A | G | 0.095408 |
| 1 | 55503448 | 0.0406795 | 0.00235743 | 1E-66 | rs17111503 | G | A | 0.268141 |
| 1 | 55516713 | -0.0168117 | 0.00295297 | 0.000000012 | rs7546522 | T | C | 0.155442 |
| 1 | 55518316 | -0.0295845 | 0.00214514 | 2.90001E-43 | rs2483205 | T | C | 0.438633 |
| 1 | 55551718 | 0.0314531 | 0.00517068 | 1.2E-09 | rs11583974 | A | G | 0.042146 |
| 1 | 55576102 | -0.0475957 | 0.00671909 | 1.39991E-12 | rs56349475 | C | T | 0.024601 |
| 1 | 55588142 | -0.0336489 | 0.00562029 | 2.1E-09 | rs79396670 | A | G | 0.035496 |
| 1 | 55453841 | -0.0538858 | 0.00722418 | 8.69961E-14 | rs146273942 | A | G | 0.023188 |
| 1 | 55492190 | -0.0283879 | 0.0023826 | 9.8992E-33 | rs2479420 | T | C | 0.73803 |
| 1 | 55496861 | -0.0294547 | 0.00507333 | 6.4E-09 | rs11810371 | A | G | 0.043743 |
| 1 | 55505647 | -0.348456 | 0.00793088 | 1E-200 | rs11591147 | T | G | 0.017468 |
| 1 | 55507649 | 0.0316517 | 0.0021463 | 3.19963E-49 | rs11206513 | T | C | 0.600617 |
| 1 | 55526428 | 0.0680285 | 0.00580615 | 1E-31 | rs11206517 | G | T | 0.033149 |
| 1 | 55448842 | 0.0177548 | 0.0025792 | 5.79963E-12 | rs2495517 | G | A | 0.794271 |
| 1 | 55470153 | -0.10344 | 0.0073736 | 1E-44 | rs12732125 | T | C | 0.020368 |
| 1 | 55484582 | 0.0125674 | 0.00221762 | 0.000000015 | rs2479395 | C | T | 0.668453 |
| 1 | 55485042 | 0.0481535 | 0.00605559 | 1.80011E-15 | rs77875082 | A | G | 0.032388 |
| 1 | 55513183 | -0.0386615 | 0.00705365 | 4.20001E-08 | rs41294821 | T | C | 0.022807 |
| 1 | 55521313 | 0.0425743 | 0.00218093 | 7.29962E-85 | rs472495 | T | G | 0.648959 |
| 1 | 55583210 | -0.192336 | 0.00997554 | 7.8001E-83 | rs530804537 | A | G | 0.011303 |
| 1 | 55466303 | -0.0187129 | 0.00324835 | 8.40001E-09 | rs55637835 | T | C | 0.120881 |
| 1 | 55496648 | -0.0202563 | 0.00254032 | 1.50003E-15 | rs12739979 | T | C | 0.246521 |
| 1 | 55500978 | 0.0509816 | 0.00777535 | 5.50047E-11 | rs72660548 | G | C | 0.018458 |
| 1 | 55518622 | -0.0340702 | 0.0048672 | 2.60016E-12 | rs45613943 | C | T | 0.048725 |

**Supplementary Table 6.** The detail of instrumental variable corresponding to APOB.

| chr.exposure | pos.exposure | beta.exposure | se.exposure | pval.exposure | SNP | effect_allele.exposure | other_allele.exposure | eaf.exposure |
| --- | --- | --- | --- | --- | --- | --- | --- | --- |
| 2 | 21147179 | 0.0598493 | 0.0032162 | 2.70023E-77 | rs4128553 | T | C | 0.119564 |
| 2 | 21208211 | -0.0527285 | 0.0024772 | 1.5E-100 | rs7557067 | G | A | 0.227783 |
| 2 | 21246382 | -0.0729131 | 0.00986922 | 1.50003E-13 | rs72653066 | G | C | 0.011361 |
| 2 | 21250223 | 0.0746047 | 0.00209394 | 1E-200 | rs11901649 | A | G | 0.443659 |
| 2 | 21334717 | -0.0959288 | 0.0099138 | 3.80014E-22 | rs114185526 | T | C | 0.011161 |
| 2 | 21349199 | -0.0189781 | 0.0021617 | 1.59993E-18 | rs312961 | C | T | 0.366218 |
| 2 | 21366682 | -0.0816866 | 0.00923295 | 8.99912E-19 | rs72782175 | C | T | 0.012867 |
| 2 | 21135220 | 0.0709235 | 0.0100731 | 1.9002E-12 | rs137978266 | G | A | 0.011587 |
| 2 | 21182116 | -0.0809843 | 0.00417288 | 6.70039E-84 | rs36134738 | G | A | 0.069376 |
| 2 | 21216112 | -0.0214817 | 0.00380881 | 0.000000017 | rs76384951 | C | A | 0.082516 |
| 2 | 21238897 | 0.0749277 | 0.00751954 | 2.19989E-23 | rs497166 | C | T | 0.980477 |
| 2 | 21255764 | -0.107563 | 0.0122418 | 1.50003E-18 | rs184507838 | T | C | 0.007496 |
| 2 | 21261998 | 0.04341 | 0.0074869 | 6.69993E-09 | rs12720796 | C | A | 0.019689 |
| 2 | 21133883 | -0.0465695 | 0.00338712 | 5.19996E-43 | rs10198175 | G | A | 0.891006 |
| 2 | 21149771 | -0.0364752 | 0.00219716 | 6.79986E-62 | rs140798831 | C | T | 0.656841 |
| 2 | 21179379 | -0.0709265 | 0.00960711 | 1.59993E-13 | rs149196850 | A | G | 0.012092 |
| 2 | 21241505 | -0.064958 | 0.00364692 | 5.70033E-71 | rs12713956 | G | A | 0.089244 |
| 2 | 21289068 | -0.0804294 | 0.00502529 | 1.20005E-57 | rs72902590 | A | G | 0.044841 |
| 2 | 21323839 | 0.0795386 | 0.00374364 | 3.6E-100 | rs377122620 | G | T | 0.112914 |
| 2 | 21205563 | 0.0282968 | 0.0022359 | 1E-36 | rs10164442 | G | A | 0.31836 |
| 2 | 21228827 | 0.0394744 | 0.00348176 | 8.60003E-30 | rs1801701 | T | C | 0.098843 |
| 2 | 21229446 | 0.0981243 | 0.0104207 | 4.70002E-21 | rs1042023 | C | G | 0.010503 |
| 2 | 21297051 | 0.0764916 | 0.00221254 | 1E-200 | rs1897083 | G | A | 0.334676 |
| 2 | 21301892 | 0.046023 | 0.00493809 | 1.20005E-20 | rs6756743 | T | C | 0.047286 |
| 2 | 21145665 | -0.0442269 | 0.00563616 | 4.30031E-15 | rs10175646 | C | T | 0.964662 |
| 2 | 21216815 | 0.065847 | 0.00215559 | 1E-200 | rs62122481 | A | C | 0.376939 |
| 2 | 21220955 | -0.0487348 | 0.00260358 | 3.50026E-78 | rs4665709 | A | G | 0.19872 |
| 2 | 21233972 | -0.115228 | 0.00533769 | 2.3014E-103 | rs533617 | C | T | 0.039479 |
| 2 | 21253628 | 0.0628605 | 0.0032695 | 2.19989E-82 | rs12720807 | T | A | 0.118098 |
| 2 | 21257927 | 0.0618146 | 0.00553677 | 6.09958E-29 | rs12720842 | C | T | 0.03649 |
| 2 | 21263639 | 0.0989323 | 0.00293061 | 1E-200 | rs531819 | G | T | 0.850253 |
| 2 | 21295065 | -0.0283419 | 0.00304812 | 1.39991E-20 | rs540156 | T | C | 0.140353 |

**Supplementary Table 7.** The detail of instrumental variable corresponding to APOC3.

| chr.exposure | pos.exposure | beta.exposure | se.exposure | pval.exposure | SNP | effect_allele.exposure | other_allele.exposure | eaf.exposure |
| --- | --- | --- | --- | --- | --- | --- | --- | --- |
| 11 | 116610294 | -0.0729835 | 0.00257629 | 1.4997E-176 | rs61905084 | C | T | 0.180713 |
| 11 | 116623659 | -0.0937927 | 0.00207286 | 1E-200 | rs180327 | T | C | 0.645279 |
| 11 | 116629555 | -0.0566905 | 0.00652029 | 3.50026E-18 | rs150233369 | A | G | 0.02459 |
| 11 | 116675294 | -0.112042 | 0.00303292 | 1E-200 | rs6589570 | T | A | 0.875877 |
| 11 | 116677048 | -0.0462866 | 0.00681198 | 1.10002E-11 | rs12802202 | C | T | 0.02272 |
| 11 | 116700169 | -0.0251343 | 0.002071 | 6.79986E-34 | rs2854116 | T | C | 0.645289 |
| 11 | 116710968 | -0.0793887 | 0.00220781 | 1E-200 | rs613808 | G | A | 0.715249 |
| 11 | 116774447 | -0.0387731 | 0.00379278 | 1.59993E-24 | rs34144542 | G | A | 0.075127 |
| 11 | 116606766 | -0.245288 | 0.00393661 | 1E-200 | rs1974718 | A | G | 0.931914 |
| 11 | 116609540 | -0.0425397 | 0.00448663 | 2.49977E-21 | rs11216122 | T | G | 0.05143 |
| 11 | 116639692 | -0.0688862 | 0.00209055 | 1E-200 | rs1268353 | T | C | 0.342494 |
| 11 | 116645275 | -0.131603 | 0.0023033 | 1E-200 | rs7118999 | T | C | 0.753003 |
| 11 | 116645336 | -0.0751594 | 0.00753946 | 2.09991E-23 | rs3087611 | T | A | 0.017798 |
| 11 | 116649131 | -0.050457 | 0.00690954 | 2.80027E-13 | rs111732554 | C | G | 0.02099 |
| 11 | 116650571 | -0.0615155 | 0.00828196 | 1.10002E-13 | rs113271699 | A | C | 0.014811 |
| 11 | 116669428 | -0.0420515 | 0.00260213 | 9.60064E-59 | rs35412484 | C | T | 0.176984 |
| 11 | 116679155 | -0.0615272 | 0.00393819 | 5.10035E-55 | rs75542613 | A | G | 0.068647 |
| 11 | 116699395 | -0.0703707 | 0.00566642 | 2.09991E-35 | rs12721078 | A | C | 0.031903 |
| 11 | 116702778 | 0.0603138 | 0.00810637 | 1E-13 | rs5132 | T | C | 0.015423 |
| 11 | 116752219 | -0.119998 | 0.00332452 | 1E-200 | rs7124741 | T | A | 0.900821 |
| 11 | 116797442 | -0.0401412 | 0.00482736 | 9.09913E-17 | rs112701434 | C | T | 0.045239 |
| 11 | 116624922 | 0.28257 | 0.0105243 | 8.5901E-159 | rs139524394 | T | C | 0.009494 |
| 11 | 116628057 | 0.209513 | 0.00392047 | 1E-200 | rs61905112 | A | G | 0.068441 |
| 11 | 116682668 | 0.131801 | 0.00826334 | 2.80027E-57 | rs149137426 | A | T | 0.015036 |
| 11 | 116699389 | -0.213096 | 0.00537509 | 1E-200 | rs10790164 | G | A | 0.966364 |
| 11 | 116730833 | -0.045801 | 0.0023951 | 1.59993E-81 | rs545274 | T | C | 0.220687 |
| 11 | 116749084 | -0.0404332 | 0.00727679 | 2.80001E-08 | rs146092979 | A | C | 0.019597 |
| 11 | 116759332 | 0.0665803 | 0.00316654 | 3.80014E-98 | rs1473177 | C | T | 0.110588 |
| 11 | 116627177 | 0.23866 | 0.0122749 | 3.29989E-84 | rs193108398 | A | G | 0.006972 |
| 11 | 116649759 | -0.0498568 | 0.00720256 | 4.49987E-12 | rs74773964 | C | T | 0.019233 |
| 11 | 116661488 | 0.110289 | 0.00638576 | 7.8001E-67 | rs3135507 | T | C | 0.024962 |
| 11 | 116674818 | -0.0512718 | 0.00211714 | 1.4997E-129 | rs1729408 | G | A | 0.34001 |
| 11 | 116676590 | -0.0459859 | 0.00735965 | 4.09996E-10 | rs4080140 | A | G | 0.018845 |
| 11 | 116714293 | 0.33152 | 0.0105893 | 1E-200 | rs141469619 | G | A | 0.009917 |
| 11 | 116635951 | -0.0342105 | 0.00529141 | 1E-10 | rs79610135 | C | T | 0.03647 |
| 11 | 116660008 | 0.289951 | 0.0119819 | 2.3014E-129 | rs142958146 | G | A | 0.007237 |
| 11 | 116667545 | -0.0970207 | 0.00456283 | 2.4998E-100 | rs75919952 | T | C | 0.050151 |
| 11 | 116693871 | 0.185471 | 0.00788062 | 1.7989E-122 | rs12721041 | T | C | 0.016054 |
| 11 | 116748314 | -0.0338415 | 0.00385233 | 1.59993E-18 | rs681524 | C | T | 0.072077 |
| 11 | 116771356 | -0.11614 | 0.00678782 | 1.29987E-65 | rs595137 | T | C | 0.977799 |

**Supplementary Table 8.** The detail of instrumental variable corresponding to LPL.

| chr.exposure | pos.exposure | beta.exposure | se.exposure | pval.exposure | SNP | effect_allele.exposure | other_allele.exposure | eaf.exposure |
| --- | --- | --- | --- | --- | --- | --- | --- | --- |
| 8 | 19692026 | -0.0200934 | 0.00199979 | 9.3994E-24 | rs2934 | C | A | 0.472139 |
| 8 | 19694420 | 0.0513883 | 0.0084517 | 1.2E-09 | rs140329088 | G | A | 0.014566 |
| 8 | 19723503 | 0.0214263 | 0.00212817 | 7.70016E-24 | rs2044061 | C | T | 0.321186 |
| 8 | 19727047 | 0.0584298 | 0.0027247 | 5.1051E-102 | rs1441778 | T | C | 0.843832 |
| 8 | 19729605 | -0.025995 | 0.00248125 | 1.10002E-25 | rs1441779 | C | T | 0.796007 |
| 8 | 19749390 | -0.0310554 | 0.00198677 | 4.49987E-55 | rs3898938 | T | C | 0.48184 |
| 8 | 19768150 | -0.149125 | 0.00841587 | 2.99985E-70 | rs142084074 | A | G | 0.015151 |
| 8 | 19813676 | 0.0250664 | 0.00277053 | 1.50003E-19 | rs270 | A | C | 0.158181 |
| 8 | 19815098 | 0.0432872 | 0.0025137 | 1.9002E-66 | rs283 | T | C | 0.199272 |
| 8 | 19815556 | -0.116913 | 0.00229945 | 1E-200 | rs287 | G | A | 0.246649 |
| 8 | 19848117 | 0.0757662 | 0.0083203 | 8.49963E-20 | rs11781692 | A | C | 0.014572 |
| 8 | 19852491 | 0.0720911 | 0.00674999 | 1.29987E-26 | rs74444445 | C | T | 0.023877 |
| 8 | 19888586 | -0.194709 | 0.00538679 | 1E-200 | rs138295898 | C | T | 0.037021 |
| 8 | 19890641 | 0.0527234 | 0.00792251 | 2.80027E-11 | rs187544997 | G | C | 0.016545 |
| 8 | 19902490 | -0.237144 | 0.0154046 | 1.80011E-53 | rs77842142 | T | C | 0.005137 |
| 8 | 19903238 | 0.0518802 | 0.00786956 | 4.30031E-11 | rs144469617 | A | G | 0.016219 |
| 8 | 19918088 | -0.142708 | 0.0077424 | 7.29962E-76 | rs142565486 | T | C | 0.016753 |
| 8 | 19672101 | 0.041441 | 0.00487016 | 1.80011E-17 | rs36164932 | T | C | 0.046886 |
| 8 | 19714837 | 0.0239806 | 0.00329184 | 3.19963E-13 | rs77312736 | A | G | 0.106044 |
| 8 | 19776981 | -0.0869921 | 0.00286436 | 1E-200 | rs4466415 | C | A | 0.140118 |
| 8 | 19777695 | -0.168548 | 0.00650586 | 5.4954E-148 | rs75218485 | T | C | 0.023587 |
| 8 | 19826373 | -0.0637172 | 0.00198763 | 1E-200 | rs2197089 | A | G | 0.549443 |
| 8 | 19835050 | -0.0465992 | 0.0025421 | 4.70002E-75 | rs2165557 | T | A | 0.186042 |
| 8 | 19838353 | -0.065242 | 0.00667819 | 1.50003E-22 | rs73600043 | T | G | 0.0225 |
| 8 | 19843748 | 0.04264 | 0.00658846 | 9.70063E-11 | rs147011441 | A | G | 0.024998 |
| 8 | 19847645 | 0.054454 | 0.00925949 | 4.09996E-09 | rs117956669 | G | T | 0.011507 |
| 8 | 19913833 | 0.0477592 | 0.00654152 | 2.90001E-13 | rs118045108 | T | C | 0.023348 |
| 8 | 19680259 | -0.0385542 | 0.00233045 | 1.80011E-61 | rs60329942 | A | G | 0.238433 |
| 8 | 19738408 | -0.0875018 | 0.00891533 | 9.70063E-23 | rs148383135 | A | G | 0.013042 |
| 8 | 19755175 | -0.0787215 | 0.00254322 | 1E-200 | rs73597688 | A | C | 0.18651 |
| 8 | 19757036 | -0.0772277 | 0.00874754 | 1.10002E-18 | rs75240547 | C | G | 0.013054 |
| 8 | 19778142 | 0.0285317 | 0.0034706 | 1.99986E-16 | rs34761945 | T | C | 0.093687 |
| 8 | 19805708 | 0.166326 | 0.00758716 | 1.5996E-106 | rs1801177 | A | G | 0.017255 |
| 8 | 19823192 | 0.154168 | 0.00606855 | 2.3014E-142 | rs3289 | C | T | 0.027497 |
| 8 | 19890654 | 0.0523227 | 0.00291856 | 7.19946E-72 | rs4557718 | C | T | 0.132042 |
| 8 | 19710468 | 0.0316643 | 0.00201037 | 6.79986E-56 | rs4244456 | C | T | 0.548311 |
| 8 | 19717091 | 0.0467802 | 0.0024477 | 1.99986E-81 | rs6586874 | G | A | 0.785063 |
| 8 | 19731858 | -0.0651127 | 0.00873024 | 8.80035E-14 | rs140801028 | G | C | 0.013779 |
| 8 | 19751560 | 0.0287463 | 0.00198414 | 1.39991E-47 | rs1441776 | G | C | 0.510501 |
| 8 | 19756813 | -0.0391045 | 0.00203781 | 4.49987E-82 | rs10102717 | T | C | 0.392711 |
| 8 | 19813529 | 0.226897 | 0.00739462 | 1E-200 | rs268 | G | A | 0.018189 |
| 8 | 19817476 | -0.159413 | 0.00698442 | 2.6002E-115 | rs308 | G | T | 0.020597 |
| 8 | 19822741 | -0.156954 | 0.00576245 | 2.3014E-163 | rs117910839 | A | T | 0.031406 |
| 8 | 19830921 | -0.171506 | 0.00299412 | 1E-200 | rs10096633 | T | C | 0.124384 |
| 8 | 19837269 | -0.0410132 | 0.00451414 | 1E-19 | rs117303935 | T | C | 0.052188 |
| 8 | 19854773 | -0.0470839 | 0.00273013 | 1.20005E-66 | rs2410622 | C | T | 0.839014 |
| 8 | 19891915 | -0.071252 | 0.00931953 | 2.09991E-14 | rs144014029 | C | T | 0.011457 |
| 8 | 19659392 | -0.0276683 | 0.00503144 | 3.79997E-08 | rs11786327 | C | A | 0.040567 |
| 8 | 19662937 | 0.0773626 | 0.00758596 | 1.99986E-24 | rs79153732 | T | C | 0.017389 |
| 8 | 19683668 | 0.0264588 | 0.00229712 | 1.10002E-30 | rs2410614 | A | G | 0.754489 |
| 8 | 19710388 | 0.0178256 | 0.00276695 | 1.2E-10 | rs4922108 | C | T | 0.15026 |
| 8 | 19727569 | 0.0346894 | 0.00463312 | 7.00003E-14 | rs17091574 | C | T | 0.049222 |
| 8 | 19774005 | 0.0377649 | 0.00666587 | 0.000000015 | rs148048657 | A | G | 0.025278 |
| 8 | 19825055 | -0.0399801 | 0.00544845 | 2.19989E-13 | rs113831503 | T | C | 0.034267 |
| 8 | 19871320 | -0.0651913 | 0.0019904 | 1E-200 | rs1372343 | T | C | 0.452231 |
| 8 | 19888313 | 0.0472318 | 0.00367803 | 9.60064E-38 | rs73208821 | C | G | 0.078857 |
| 8 | 19910576 | -0.0531426 | 0.00603306 | 1.29987E-18 | rs148754782 | A | G | 0.027908 |
| 8 | 19912060 | -0.0807274 | 0.00219557 | 1E-200 | rs66462329 | A | G | 0.283325 |

**Supplementary Table 9.** The effect of inhibitors on CHD.

| Outcome | Target | method | nsnp | pval | or | or_lci95 | or_uci95 |
| --- | --- | --- | --- | --- | --- | --- | --- |
| CHD | LDLR | Weighted median | 42 | 1.98E-19 | 0.46532448 | 0.298983803 | 0.631665157 |
|  |  | Inverse variance weighted | 42 | 5.63E-29 | 0.438426546 | 0.293759338 | 0.583093755 |
|  |  | Weighted mode | 42 | 5.90E-08 | 0.471199862 | 0.248028067 | 0.694371658 |
|  | HMGCR | Weighted median | 19 | 6.48E-05 | 0.620398039 | 0.386163217 | 0.854632862 |
|  |  | Inverse variance weighted | 19 | 8.29E-08 | 0.618313896 | 0.442533946 | 0.794093846 |
|  |  | Weighted mode | 19 | 0.000434125 | 0.622638086 | 0.406519412 | 0.838756759 |
|  | NPC1L1 | Weighted median | 6 | 0.008157355 | 0.497667712 | -0.01933831 | 1.014673735 |
|  |  | Inverse variance weighted | 6 | 0.000492408 | 0.457458451 | 0.017596075 | 0.897320826 |
|  |  | Weighted mode | 6 | 0.090962158 | 0.528688635 | -0.069177182 | 1.126554452 |
|  | PCSK9 | Weighted median | 28 | 7.62E-11 | 0.470063786 | 0.242715472 | 0.697412101 |
|  |  | Inverse variance weighted | 28 | 7.20E-24 | 0.435496462 | 0.27376347 | 0.597229454 |
|  |  | Weighted mode | 28 | 7.65E-07 | 0.471939431 | 0.241565155 | 0.702313708 |
|  | APOB | Weighted median | 29 | 0.000104193 | 0.735256621 | 0.579928162 | 0.89058508 |
|  |  | Inverse variance weighted | 29 | 4.60E-07 | 0.713223329 | 0.581858963 | 0.844587696 |
|  |  | Weighted mode | 29 | 0.005270208 | 0.749811312 | 0.563295421 | 0.936327203 |
|  | APOC3 | Weighted median | 37 | 0.000317669 | 0.857698513 | 0.77413526 | 0.941261765 |
|  |  | Inverse variance weighted | 37 | 1.22E-12 | 0.816773993 | 0.76092771 | 0.872620277 |
|  |  | Weighted mode | 37 | 0.000101399 | 0.852405497 | 0.780762458 | 0.924048536 |
|  | LPL | Weighted median | 56 | 1.77E-15 | 0.662096691 | 0.560515529 | 0.763677854 |
|  |  | Inverse variance weighted | 56 | 1.79E-40 | 0.6414447 | 0.57610202 | 0.70678738 |
|  |  | Weighted mode | 56 | 4.63E-09 | 0.671192174 | 0.558680853 | 0.783703495 |

**Supplementary Table 10.** The effect of inhibitors on ED.

| Outcome | Target | method | nsnp | pval | or | or_lci95 | or_uci95 |
| --- | --- | --- | --- | --- | --- | --- | --- |
| ED (FinnnGen database) | LDLR | Inverse variance weighted | 46 | 0.4404197 | 0.8344691 | 0.3747314 | 1.2942069 |
|  |  | Weighted median | 46 | 0.7671081 | 1.1086196 | 0.426195 | 1.7910442 |
|  |  | Weighted mode | 46 | 0.5715131 | 1.2224584 | 0.5317676 | 1.9131492 |
|  | HMGCR | Inverse variance weighted | 19 | 0.1290357 | 1.7681358 | 1.0322223 | 2.5040493 |
|  |  | Weighted median | 19 | 0.3269186 | 1.6303917 | 0.6530896 | 2.6076937 |
|  |  | Weighted mode | 19 | 0.2971117 | 1.7152159 | 0.7303685 | 2.7000633 |
|  | NPC1L1 | Inverse variance weighted | 6 | 0.3885479 | 2.1195608 | 0.4119782 | 3.8271433 |
|  |  | Weighted median | 6 | 0.6742339 | 1.5440009 | -0.481431 | 3.5694331 |
|  |  | Weighted mode | 6 | 0.8084853 | 1.3274967 | -0.845385 | 3.5003787 |
|  | PCSK9 | Inverse variance weighted | 29 | 0.7587853 | 0.9334436 | 0.493831 | 1.3730561 |
|  |  | Weighted median | 29 | 0.4954999 | 0.8180176 | 0.2403837 | 1.3956515 |
|  |  | Weighted mode | 29 | 0.5240935 | 0.8358622 | 0.291149 | 1.3805753 |
|  | APOB | Inverse variance weighted | 31 | 3.32E-05 | 2.7697326 | 2.2886246 | 3.2508406 |
|  |  | Weighted median | 31 | 9.604E-05 | 3.0971146 | 2.5290349 | 3.6651944 |
|  |  | Weighted mode | 31 | 0.0009672 | 3.1824272 | 2.5622092 | 3.8026451 |
|  | APOC3 | Inverse variance weighted | 57 | 0.6348861 | 0.931889 | 0.6407269 | 1.2230511 |
|  |  | Weighted median | 57 | 0.511274 | 1.152426 | 0.7291002 | 1.5757517 |
|  |  | Weighted mode | 57 | 0.6794125 | 1.0922614 | 0.6758995 | 1.5086233 |
|  | LPL | Inverse variance weighted | 39 | 0.1608176 | 0.837467 | 0.5895563 | 1.0853777 |
|  |  | Weighted median | 39 | 0.4818838 | 0.8814329 | 0.5297011 | 1.2331647 |
|  |  | Weighted mode | 39 | 0.6525621 | 0.9261775 | 0.5949413 | 1.2574137 |
| ED (ebi database) | LDLR | Inverse variance weighted | 47 | 0.0048478 | 0.7545852 | 0.5586622 | 0.9505082 |
|  |  | Weighted median | 47 | 0.2752257 | 0.8516236 | 0.5631123 | 1.1401349 |
|  |  | Weighted mode | 47 | 0.5225973 | 0.8959146 | 0.5615477 | 1.2302815 |
|  | HMGCR | Inverse variance weighted | 19 | 0.06285 | 0.7159882 | 0.3639822 | 1.0679942 |
|  |  | Weighted median | 19 | 0.4245901 | 0.8214342 | 0.3385954 | 1.304273 |
|  |  | Weighted mode | 19 | 0.2815769 | 0.7620115 | 0.2821199 | 1.2419031 |
|  | NPC1L1 | Inverse variance weighted | 6 | 0.6848285 | 1.1913687 | 0.3458015 | 2.0369358 |
|  |  | Weighted median | 6 | 0.8705986 | 0.9111923 | -0.207805 | 2.03019 |
|  |  | Weighted mode | 6 | 0.8554762 | 0.8816337 | -0.406022 | 2.1692891 |
|  | PCSK9 | Inverse variance weighted | 33 | 0.4099606 | 1.1357649 | 0.8329352 | 1.4385946 |
|  |  | Weighted median | 33 | 0.0684845 | 1.4580699 | 1.05235 | 1.8637897 |
|  |  | Weighted mode | 33 | 0.1065983 | 1.4121878 | 1.0047772 | 1.8195984 |
|  | APOB | Inverse variance weighted | 32 | 0.8712093 | 0.980294 | 0.7396771 | 1.2209108 |
|  |  | Weighted median | 32 | 0.1022242 | 0.7737341 | 0.4660584 | 1.0814099 |
|  |  | Weighted mode | 32 | 0.172593 | 0.768519 | 0.3988921 | 1.138146 |
|  | APOC3 | Inverse variance weighted | 58 | 0.1379688 | 0.9056632 | 0.7747395 | 1.036587 |
|  |  | Weighted median | 58 | 0.6695571 | 0.9603901 | 0.7747692 | 1.146011 |
|  |  | Weighted mode | 58 | 0.6473361 | 0.9579281 | 0.7747457 | 1.1411104 |
|  | LPL | Inverse variance weighted | 39 | 0.0867079 | 0.8943691 | 0.7666371 | 1.022101 |
|  |  | Weighted median | 39 | 0.3518698 | 0.9202677 | 0.745334 | 1.0952013 |
|  |  | Weighted mode | 39 | 0.2672608 | 0.9091433 | 0.7433276 | 1.074959 |

**Supplementary Table 11.** The result of heterogeneity test and horizontal pleiotropic test.

| Outcomes | Drug Target | Heterogeneity test | | | | Horizontal pleiotropic test | | |
| --- | --- | --- | --- | --- | --- | --- | --- | --- |
|  |  | Method | Q | Q_df | Q_pval | egger_intercept | SE | *p*-value |
| ED (FinnnGen database) | LDLR | MR Egger | 48.48101 | 44 | 0.2970437 | -0.01259084 | 0.01960911 | 0.5241451 |
|  |  | Inverse variance weighted | 48.93528 | 45 | 0.3180515 |  |  |  |
|  | HMGCR | MR Egger | 8.361030 | 17 | 0.9582057 | -0.02058263 | 0.05162146 | 0.6950615 |
|  |  | Inverse variance weighted | 8.520009 | 18 | 0.9698478 |  |  |  |
|  | NPC1L1 | MR Egger | 0.8706588 | 4 | 0.9287320 | -0.0447066 | 0.06725536 | 0.5425872 |
|  |  | Inverse variance weighted | 1.3125236 | 5 | 0.9336377 |  |  |  |
|  | PCSK9 | MR Egger | 24.86569 | 27 | 0.5819886 | -0.006478661 | 0.01752797 | 0.7145501 |
|  |  | Inverse variance weighted | 25.00231 | 28 | 0.6277095 |  |  |  |
|  | APOB | MR Egger | 38.99282 | 29 | 0.10180162 | -0.04873507 | 0.03666721 | 0.1941715 |
|  |  | Inverse variance weighted | 41.36810 | 30 | 0.08096947 |  |  |  |
|  | APOC3 | MR Egger | 22.38461 | 37 | 0.9722008 | 0.05101332 | 0.02387445 | 0.03929792 |
|  |  | Inverse variance weighted | 26.95024 | 38 | 0.9095552 |  |  |  |
|  | LPL | MR Egger | 39.24367 | 55 | 0.9463648 | 0.03154351 | 0.01625496 | 0.057446 |
|  |  | Inverse variance weighted | 43.00940 | 56 | 0.8985655 |  |  |  |
| ED (ebi database) | LDLR | MR Egger | 29.02214 | 45 | 0.9689936 | 0.01208562 | 0.008622099 | 0.1678642 |
|  |  | Inverse variance weighted | 30.98691 | 46 | 0.9560034 |  |  |  |
|  | HMGCR | MR Egger | 13.0442 | 17 | 0.7332221 | -0.01227406 | 0.02486313 | 0.62786 |
|  |  | Inverse variance weighted | 13.28791 | 18 | 0.7741933 |  |  |  |
|  | NPC1L1 | MR Egger | 3.360264 | 4 | 0.4994409 | -0.01202152 | 0.03198559 | 0.7261177 |
|  |  | Inverse variance weighted | 3.501521 | 5 | 0.6231575 |  |  |  |
|  | PCSK9 | MR Egger | 34.62347 | 31 | 0.2989626 | 0.02225053 | 0.009957979 | 0.03280168 |
|  |  | Inverse variance weighted | 40.19979 | 32 | 0.1514179 |  |  |  |
|  | APOB | MR Egger | 39.00056 | 30 | 0.1257161 | 0.03861686 | 0.01700008 | 0.0304494 |
|  |  | Inverse variance weighted | 45.7087 | 31 | 0.04305349 |  |  |  |
|  | APOC3 | MR Egger | 44.93086 | 37 | 0.173723 | 0.003898422 | 0.01186632 | 0.7443644 |
|  |  | Inverse variance weighted | 45.06192 | 38 | 0.2004115 |  |  |  |
|  | LPL | MR Egger | 57.66206 | 56 | 0.4135523 | 0.009317895 | 0.007520588 | 0.2205191 |
|  |  | Inverse variance weighted | 59.24271 | 57 | 0.3937167 |  |  |  |

**Supplementary Table 12.** The result of heterogeneity test and horizontal pleiotropic test (adjusted).

| Outcomes | Drug Target | Heterogeneity test | | | | Horizontal pleiotropic test | | |
| --- | --- | --- | --- | --- | --- | --- | --- | --- |
|  |  | Method | Q | Q_df | Q_pval | egger_intercept | SE | p-value |
| ED (FinnnGen database) | APOC3 | MR Egger | 17.44333 | 28 | 0.9393111 | 0.0420429 | 0.0287549 | 0.1548442 |
|  |  | Inverse variance weighted | 19.58111 | 29 | 0.9054705 |  |  |  |
| ED (ebi database) | APOB | MR Egger | 23.41321 | 17 | 0.1362755 | 0.0274135 | 0.0424424 | 0.5269682 |
|  |  | Inverse variance weighted | 23.98778 | 18 | 0.1554286 |  |  |  |
|  | PCSK9 | MR Egger | 26.18853 | 27 | 0.508157 | 0.01686801 | 0.010348 | 0.1146989 |
|  |  | Inverse variance weighted | 28.84566 | 28 | 0.4203872 |  |  |  |

**Supplementary Table 13.** The effect of inhibitors on sex hormones.

| Outcome | Target | method | nsnp | pval | or | or_lci95 | or_uci95 |
| --- | --- | --- | --- | --- | --- | --- | --- |
| TT | LDLR | Inverse variance weighted | 48 | 0.001737164 | 1.048100946 | 1.018699474 | 1.077502417 |
|  |  | Weighted median | 48 | 0.002462296 | 1.06692901 | 1.024993689 | 1.10886433 |
|  |  | Weighted mode | 48 | 0.022244535 | 1.063334782 | 1.012427097 | 1.114242467 |
|  | HMGCR | Inverse variance weighted | 19 | 7.64E-14 | 0.79044618 | 0.728796749 | 0.852095611 |
|  |  | Weighted median | 19 | 1.64E-07 | 0.810437343 | 0.731758899 | 0.889115788 |
|  |  | Weighted mode | 19 | 1.37E-05 | 0.796772932 | 0.721351067 | 0.872194798 |
|  | PCSK9 | Inverse variance weighted | 33 | 0.091228375 | 1.034110294 | 0.995186058 | 1.07303453 |
|  |  | Weighted median | 33 | 0.022921944 | 1.068839046 | 1.011477069 | 1.126201023 |
|  |  | Weighted mode | 33 | 0.087177425 | 1.058920962 | 0.995330556 | 1.122511368 |
|  | LPL | Inverse variance weighted | 58 | 7.48E-14 | 1.080325181 | 1.060077588 | 1.100572773 |
|  |  | Weighted median | 58 | 2.38E-06 | 1.076180987 | 1.045681399 | 1.106680575 |
|  |  | Weighted mode | 58 | 2.28E-05 | 1.075396217 | 1.044520377 | 1.106272057 |
| BT | LDLR | Inverse variance weighted | 48 | 0.860561365 | 1.002771933 | 0.971885647 | 1.033658219 |
|  |  | Weighted median | 48 | 0.489532323 | 0.984369893 | 0.939688872 | 1.029050915 |
|  |  | Weighted mode | 48 | 0.713782128 | 0.990468726 | 0.939599607 | 1.041337846 |
|  | HMGCR | Inverse variance weighted | 19 | 2.24E-10 | 0.823701118 | 0.763782711 | 0.883619526 |
|  |  | Weighted median | 19 | 3.07E-05 | 0.843563566 | 0.763570242 | 0.92355689 |
|  |  | Weighted mode | 19 | 0.000335833 | 0.834239037 | 0.753740855 | 0.914737218 |
|  | NPC1L1 | Inverse variance weighted | 6 | 0.044795363 | 1.159530317 | 1.014950609 | 1.304110025 |
|  |  | Weighted median | 6 | 0.100779428 | 1.158724618 | 0.982775915 | 1.33467332 |
|  |  | Weighted mode | 6 | 0.243472748 | 1.16131684 | 0.939556416 | 1.383077264 |
|  | PCSK9 | Inverse variance weighted | 6 | 0.044795363 | 1.159530317 | 1.014950609 | 1.304110025 |
|  |  | Weighted median | 6 | 0.100779428 | 1.158724618 | 0.982775915 | 1.33467332 |
|  |  | Weighted mode | 6 | 0.243472748 | 1.16131684 | 0.939556416 | 1.383077264 |
|  | APOB | Inverse variance weighted | 32 | 0.160864793 | 0.978068877 | 0.94707164 | 1.009066113 |
|  |  | Weighted median | 32 | 0.334947414 | 0.979319422 | 0.936839558 | 1.021799285 |
|  |  | Weighted mode | 32 | 0.415368503 | 0.981048725 | 0.935623191 | 1.026474259 |
| E2 | LDLR | Inverse variance weighted | 48 | 0.897849013 | 0.993313137 | 0.890879999 | 1.095746274 |
|  |  | Weighted median | 48 | 0.905103699 | 0.99123917 | 0.846570766 | 1.135907575 |
|  |  | Weighted mode | 48 | 0.999173992 | 1.000082362 | 0.844982122 | 1.155182603 |
|  | HMGCR | Inverse variance weighted | 19 | 0.41576548 | 1.085635086 | 0.887741568 | 1.283528605 |
|  |  | Weighted median | 19 | 0.348514694 | 1.133919422 | 0.871156895 | 1.396681949 |
|  |  | Weighted mode | 19 | 0.464501797 | 1.110692702 | 0.835363489 | 1.386021916 |
|  | NPC1L1 | Inverse variance weighted | 6 | 0.26517517 | 0.760689568 | 0.279538771 | 1.241840365 |
|  |  | Weighted median | 6 | 0.566388875 | 0.847220804 | 0.280480925 | 1.413960682 |
|  |  | Weighted mode | 6 | 0.7308675 | 0.882393774 | 0.208361085 | 1.556426464 |
|  | PCSK9 | Inverse variance weighted | 33 | 0.152243676 | 1.103780139 | 0.968599217 | 1.238961061 |
|  |  | Weighted median | 33 | 0.76705549 | 0.970753896 | 0.774360647 | 1.167147145 |
|  |  | Weighted mode | 33 | 0.994557328 | 0.999296241 | 0.798588278 | 1.200004205 |
|  | APOB | Inverse variance weighted | 32 | 0.494243499 | 1.036465065 | 0.933770781 | 1.139159349 |
|  |  | Weighted median | 32 | 0.577877002 | 1.042943405 | 0.89485057 | 1.191036241 |
|  |  | Weighted mode | 32 | 0.528356287 | 1.049578259 | 0.900852487 | 1.198304032 |
|  | APOC3 | Inverse variance weighted | 40 | 0.379076212 | 0.970909359 | 0.905125526 | 1.036693193 |
|  |  | Weighted median | 40 | 0.453870692 | 0.965279877 | 0.872805958 | 1.057753796 |
|  |  | Weighted mode | 40 | 0.371218168 | 0.959681977 | 0.870517478 | 1.048846476 |
| SHBG | LDLR | Inverse variance weighted | 48 | 0.002748463 | 1.047033599 | 1.016951122 | 1.077116077 |
|  |  | Weighted median | 48 | 0.030353836 | 1.049343247 | 1.005748272 | 1.092938222 |
|  |  | Weighted mode | 48 | 0.063205445 | 1.049166225 | 0.999727102 | 1.098605348 |
|  | HMGCR | Inverse variance weighted | 19 | 0.001430757 | 0.910204279 | 0.852366324 | 0.968042234 |
|  |  | Weighted median | 19 | 0.007864853 | 0.899296896 | 0.821022736 | 0.977571057 |
|  |  | Weighted mode | 19 | 0.016509162 | 0.896206742 | 0.814960884 | 0.977452599 |
|  | PCSK9 | Inverse variance weighted | 33 | 5.38E-06 | 1.09676991 | 1.056974466 | 1.136565354 |
|  |  | Weighted median | 33 | 0.027818429 | 1.069107679 | 1.009568854 | 1.128646504 |
|  |  | Weighted mode | 33 | 0.027371224 | 1.072903151 | 1.013246839 | 1.132559462 |
|  | APOC3 | Inverse variance weighted | 40 | 0.00071449 | 1.033435811 | 1.014386394 | 1.052485228 |
|  |  | Weighted median | 40 | 0.025182968 | 1.029621264 | 1.004062961 | 1.055179566 |
|  |  | Weighted mode | 40 | 0.018257567 | 1.030966771 | 1.006706859 | 1.055226682 |
|  | LPL | Inverse variance weighted | 58 | 7.09E-16 | 1.089016027 | 1.068302478 | 1.109729575 |
|  |  | Weighted median | 58 | 1.23E-07 | 1.087302022 | 1.056283222 | 1.118320821 |
|  |  | Weighted mode | 58 | 3.38E-06 | 1.086811449 | 1.055123656 | 1.118499242 |

**Supplementary Table 14.** The result of heterogeneity test and horizontal pleiotropic test.

| Outcomes | Drug Target | Heterogeneity test | | | | Horizontal pleiotropic test | | |
| --- | --- | --- | --- | --- | --- | --- | --- | --- |
|  |  | Method | Q | Q_df | Q_pval | egger_intercept | SE | p-value |
| TT | LDLR | MR Egger | 36.37569 | 46 | 0.8443588 | -5.43E-05 | 0.001322952 | 0.9674234 |
|  |  | Inverse variance weighted | 36.37738 | 47 | 0.868838 |  |  |  |
|  | HMGCR | MR Egger | 19.60951 | 17 | 0.2946875 | -0.005334939 | 0.004248899 | 0.2262436 |
|  |  | Inverse variance weighted | 21.42806 | 18 | 0.2583694 |  |  |  |
|  | PCSK9 | MR Egger | 22.64848 | 31 | 0.8616005 | 0.000599626 | 0.001330457 | 0.655348 |
|  |  | Inverse variance weighted | 22.8516 | 32 | 0.8829192 |  |  |  |
|  | LPL | MR Egger | 48.80709 | 56 | 0.7413182 | 0.000368505 | 0.0011772 | 0.7554167 |
|  |  | Inverse variance weighted | 48.90509 | 57 | 0.7685529 |  |  |  |
| BT | LDLR | MR Egger | 31.41132 | 46 | 0.9503836 | 0.000162261 | 0.001389594 | 0.9075515 |
|  |  | Inverse variance weighted | 31.42495 | 47 | 0.9606154 |  |  |  |
|  | HMGCR | MR Egger | 18.28767 | 17 | 0.3709137 | -0.000874344 | 0.004312201 | 0.8417292 |
|  |  | Inverse variance weighted | 18.3319 | 18 | 0.4339978 |  |  |  |
|  | NPC1L1 | MR Egger | 2.53231 | 4 | 0.6388596 | -0.001926011 | 0.005409241 | 0.7397817 |
|  |  | Inverse variance weighted | 2.659089 | 5 | 0.7523682 |  |  |  |
|  | PCSK9 | MR Egger | 22.64848 | 31 | 0.8616005 | 0.000599626 | 0.001330457 | 0.655348 |
|  |  | Inverse variance weighted | 22.8516 | 32 | 0.8829192 |  |  |  |
|  | APOB | MR Egger | 13.49595 | 30 | 0.9958587 | -0.000410556 | 0.002463061 | 0.868736 |
|  |  | Inverse variance weighted | 13.52373 | 31 | 0.9972886 |  |  |  |
| E2 | LDLR | MR Egger | 19.1189 | 46 | 0.9998416 | 0.004149156 | 0.004609549 | 0.3727437 |
|  |  | Inverse variance weighted | 19.92912 | 47 | 0.9998199 |  |  |  |
|  | HMGCR | MR Egger | 7.864066 | 17 | 0.9693674 | 0.01503041 | 0.01383113 | 0.2923346 |
|  |  | Inverse variance weighted | 9.045002 | 18 | 0.9586911 |  |  |  |
|  | NPC1L1 | MR Egger | 0.7522758 | 4 | 0.9447292 | 0.02620998 | 0.01799035 | 0.2188724 |
|  |  | Inverse variance weighted | 2.8748079 | 5 | 0.7192794 |  |  |  |
|  | PCSK9 | MR Egger | 26.85326 | 31 | 0.6795242 | 0.006919417 | 0.004621811 | 0.1444749 |
|  |  | Inverse variance weighted | 29.09463 | 32 | 0.6143535 |  |  |  |
|  | APOB | MR Egger | 30.18293 | 30 | 0.4563137 | 0.00554124 | 0.008199959 | 0.5043666 |
|  |  | Inverse variance weighted | 30.64237 | 31 | 0.4843333 |  |  |  |
|  | APOC3 | MR Egger | 24.22375 | 38 | 0.9596444 | 0.003480242 | 0.005910048 | 0.5594338 |
|  |  | Inverse variance weighted | 24.57052 | 39 | 0.9653361 |  |  |  |
| SHBG | LDLR | MR Egger | 27.46838 | 46 | 0.9862758 | 0.000253705 | 0.001353409 | 0.8521273 |
|  |  | Inverse variance weighted | 27.50352 | 47 | 0.9896474 |  |  |  |
|  | HMGCR | MR Egger | 8.453662 | 17 | 0.9558607 | -0.003712613 | 0.004050106 | 0.3721369 |
|  |  | Inverse variance weighted | 9.293947 | 18 | 0.9525395 |  |  |  |
|  | PCSK9 | MR Egger | 19.71384 | 31 | 0.9417422 | -0.002668378 | 0.001360111 | 0.05880862 |
|  |  | Inverse variance weighted | 23.56282 | 32 | 0.8598024 |  |  |  |
|  | APOC3 | MR Egger | 36.42755 | 38 | 0.5422448 | -0.001215122 | 0.001721514 | 0.4845897 |
|  |  | Inverse variance weighted | 36.92576 | 39 | 0.5648371 |  |  |  |
|  | LPL | MR Egger | 55.66324 | 56 | 0.4875574 | 0.000326086 | 0.001204623 | 0.7876203 |
|  |  | Inverse variance weighted | 55.73652 | 57 | 0.5225741 |  |  |  |

**Supplementary Table 15.** The effect of inhibitors on male diseases.

| Outcome | Target | method | nsnp | pval | or | or_lci95 | or_uci95 |
| --- | --- | --- | --- | --- | --- | --- | --- |
| PH | LDLR | Inverse variance weighted | 46 | 0.000260431 | 1.361640149 | 1.195958551 | 1.527321746 |
|  |  | Weighted median | 46 | 0.00181853 | 1.48438676 | 1.236115112 | 1.732658407 |
|  |  | Weighted mode | 46 | 0.007147017 | 1.449777884 | 1.191490745 | 1.708065023 |
|  | HMGCR | Inverse variance weighted | 19 | 2.69E-01 | 0.855408073 | 0.578492993 | 1.132323154 |
|  |  | Weighted median | 19 | 2.88E-01 | 0.81580249 | 0.440042086 | 1.191562894 |
|  |  | Weighted mode | 19 | 4.69E-01 | 0.866540134 | 0.48715531 | 1.245924958 |
|  | NPC1L1 | Inverse variance weighted | 6 | 0.333388027 | 1.374600879 | 0.729927573 | 2.019274186 |
|  |  | Weighted median | 6 | 0.71286092 | 1.158978914 | 0.373206656 | 1.944751173 |
|  |  | Weighted mode | 6 | 0.83556064 | 1.107672619 | 0.191031766 | 2.024313471 |
|  | PCSK9 | Inverse variance weighted | 29 | 0.006115666 | 1.260987141 | 1.095197784 | 1.426776497 |
|  |  | Weighted median | 29 | 0.136820149 | 1.185048423 | 0.961369218 | 1.408727629 |
|  |  | Weighted mode | 29 | 0.061224002 | 1.214415667 | 1.019180126 | 1.409651208 |
|  | APOB | Inverse variance weighted | 31 | 0.470561041 | 0.944808025 | 0.79059341 | 1.09902264 |
|  |  | Weighted median | 31 | 0.734661575 | 0.962116121 | 0.738780368 | 1.185451874 |
|  |  | Weighted mode | 31 | 0.661552347 | 0.947763367 | 0.709936881 | 1.185589852 |
|  | APOC3 | Inverse variance weighted | 57 | 0.062344814 | 1.109698618 | 1.000239714 | 1.219157522 |
|  |  | Weighted median | 57 | 0.097173828 | 1.14787512 | 0.984912223 | 1.310838017 |
|  |  | Weighted mode | 57 | 0.158422728 | 1.128622101 | 0.962718553 | 1.294525648 |
|  | LPL | Inverse variance weighted | 57 | 6.23E-02 | 1.109698618 | 1.000239714 | 1.219157522 |
|  |  | Weighted median | 57 | 1.03E-01 | 1.14787512 | 0.981964005 | 1.313786235 |
|  |  | Weighted mode | 57 | 1.82E-01 | 1.128622101 | 0.953203033 | 1.304041168 |
| PI | LDLR | Inverse variance weighted | 46 | 0.637202731 | 0.918812688 | 0.566916278 | 1.270709099 |
|  |  | Weighted median | 46 | 0.217242469 | 0.718555825 | 0.193548511 | 1.243563139 |
|  |  | Weighted mode | 46 | 0.254649184 | 0.70346812 | 0.105998602 | 1.300937639 |
|  | HMGCR | Inverse variance weighted | 19 | 9.09E-02 | 0.593974673 | -0.009900191 | 1.197849538 |
|  |  | Weighted median | 19 | 2.28E-01 | 0.604382342 | -0.2141982 | 1.422962885 |
|  |  | Weighted mode | 19 | 3.12E-01 | 0.660445579 | -0.121524555 | 1.442415713 |
|  | NPC1L1 | Inverse variance weighted | 6 | 0.460305764 | 1.673935712 | 0.306349908 | 3.041521517 |
|  |  | Weighted median | 6 | 0.492172136 | 1.78815754 | 0.129701317 | 3.446613763 |
|  |  | Weighted mode | 6 | 0.545233113 | 1.760140137 | 0.051450967 | 3.468829307 |
|  | APOB | Inverse variance weighted | 31 | 0.455593301 | 0.88288372 | 0.555670678 | 1.210096763 |
|  |  | Weighted median | 31 | 0.422550836 | 0.835033669 | 0.394443245 | 1.275624093 |
|  |  | Weighted mode | 31 | 0.407910287 | 0.818973331 | 0.352638752 | 1.285307909 |
|  | APOC3 | Inverse variance weighted | 57 | 0.087431266 | 1.235326553 | 0.992967553 | 1.477685553 |
|  |  | Weighted median | 57 | 0.278848949 | 1.217078999 | 0.861509504 | 1.572648493 |
|  |  | Weighted mode | 57 | 0.319964696 | 1.206180725 | 0.840020944 | 1.572340507 |
|  | LPL | Inverse variance weighted | 57 | 8.74E-02 | 1.235326553 | 0.992967553 | 1.477685553 |
|  |  | Weighted median | 57 | 2.89E-01 | 1.217078999 | 0.853756601 | 1.580401396 |
|  |  | Weighted mode | 57 | 3.50E-01 | 1.206180725 | 0.816517472 | 1.595843979 |
| PCa | LDLR | Inverse variance weighted | 48 | 0.468498609 | 1.047175723 | 0.922542485 | 1.171808961 |
|  |  | Weighted median | 48 | 0.310246073 | 1.098729073 | 0.916861505 | 1.28059664 |
|  |  | Weighted mode | 48 | 0.165157442 | 1.151681357 | 0.955355305 | 1.348007409 |
|  | HMGCR | Inverse variance weighted | 19 | 1.24E-05 | 0.616230642 | 0.399139064 | 0.83332222 |
|  |  | Weighted median | 19 | 1.06E-03 | 0.623141671 | 0.339919887 | 0.906363456 |
|  |  | Weighted mode | 19 | 3.40E-03 | 0.616841043 | 0.33597688 | 0.897705207 |
|  | NPC1L1 | Inverse variance weighted | 6 | 0.011639584 | 0.426778944 | -0.234732539 | 1.088290427 |
|  |  | Weighted median | 6 | 0.009767699 | 0.366992901 | -0.393366944 | 1.127352747 |
|  |  | Weighted mode | 6 | 0.062822302 | 0.318357822 | -0.622500644 | 1.259216288 |
|  | PCSK9 | Inverse variance weighted | 33 | 0.620995403 | 1.047310397 | 0.864069417 | 1.230551377 |
|  |  | Weighted median | 33 | 0.763679364 | 1.037546546 | 0.797258251 | 1.277834841 |
|  |  | Weighted mode | 33 | 0.789134281 | 1.032380429 | 0.800777545 | 1.263983313 |
|  | APOB | Inverse variance weighted | 31 | 0.405199548 | 0.939068671 | 0.791035318 | 1.087102023 |
|  |  | Weighted median | 31 | 0.531090397 | 0.939643991 | 0.744833208 | 1.134454774 |
|  |  | Weighted mode | 31 | 0.659548786 | 0.951762749 | 0.733983033 | 1.169542465 |
| AS | LDLR | Inverse variance weighted | 46 | 0.003420495 | 0.473013755 | -0.028259067 | 0.974286578 |
|  |  | Weighted median | 46 | 0.018554077 | 0.430334128 | -0.271619323 | 1.132287578 |
|  |  | Weighted mode | 46 | 0.019499881 | 0.410506042 | -0.309852265 | 1.13086435 |
|  | HMGCR | Inverse variance weighted | 19 | 7.96E-01 | 1.116126771 | 0.283789427 | 1.948464114 |
|  |  | Weighted median | 19 | 8.96E-01 | 0.929442828 | -0.16396946 | 2.022855117 |
|  |  | Weighted mode | 19 | 8.70E-01 | 0.904112063 | -0.282427927 | 2.090652053 |
|  | NPC1L1 | Inverse variance weighted | 6 | 0.888857454 | 1.16948632 | -1.026338017 | 3.365310656 |
|  |  | Weighted median | 6 | 0.768212303 | 0.692112607 | -1.755320319 | 3.139545534 |
|  |  | Weighted mode | 6 | 0.642626707 | 0.503961174 | -2.218100734 | 3.226023083 |
|  | PCSK9 | Inverse variance weighted | 29 | 0.1176834 | 0.665218626 | 0.15455264 | 1.175884612 |
|  |  | Weighted median | 29 | 0.181316153 | 0.635311498 | -0.029852781 | 1.300475776 |
|  |  | Weighted mode | 29 | 0.1279493 | 0.608233911 | -0.012980393 | 1.229448214 |
|  | APOB | Inverse variance weighted | 31 | 0.06306914 | 0.644623262 | 0.181604413 | 1.107642111 |
|  |  | Weighted median | 31 | 0.204192827 | 0.664301943 | 0.032909812 | 1.295694073 |
|  |  | Weighted mode | 31 | 0.290730023 | 0.704030992 | 0.06448092 | 1.343581063 |
|  | APOC3 | Inverse variance weighted | 57 | 0.06780994 | 1.357106199 | 1.029391334 | 1.684821064 |
|  |  | Weighted median | 57 | 0.051074409 | 1.595108003 | 1.125977454 | 2.064238551 |
|  |  | Weighted mode | 57 | 0.099011439 | 1.487676532 | 1.023577554 | 1.95177551 |
|  | LPL | Inverse variance weighted | 57 | 6.78E-02 | 1.357106199 | 1.029391334 | 1.684821064 |
|  |  | Weighted median | 57 | 4.88E-02 | 1.595108003 | 1.130634955 | 2.059581051 |
|  |  | Weighted mode | 57 | 9.02E-02 | 1.487676532 | 1.036130074 | 1.93922299 |
| MI | LDLR | Inverse variance weighted | 46 | 0.003941124 | 0.411603739 | -0.191927137 | 1.015134615 |
|  |  | Weighted median | 46 | 0.118991359 | 0.51447046 | -0.321085581 | 1.350026501 |
|  |  | Weighted mode | 46 | 0.154420851 | 0.511365012 | -0.396161617 | 1.418891641 |
|  | HMGCR | Inverse variance weighted | 19 | 4.72E-05 | 7.239113466 | 6.285634255 | 8.192592676 |
|  |  | Weighted median | 19 | 2.79E-03 | 7.917180841 | 6.560778657 | 9.273583026 |
|  |  | Weighted mode | 19 | 7.13E-03 | 8.08856653 | 6.738397722 | 9.438735338 |
|  | NPC1L1 | Inverse variance weighted | 6 | 0.27101667 | 3.627947125 | 1.333287822 | 5.922606428 |
|  |  | Weighted median | 6 | 0.302661115 | 4.022087898 | 1.375522365 | 6.66865343 |
|  |  | Weighted mode | 6 | 0.370899581 | 4.487061067 | 1.492809938 | 7.481312197 |
|  | PCSK9 | Inverse variance weighted | 29 | 0.605534306 | 0.862291868 | 0.300007468 | 1.424576267 |
|  |  | Weighted median | 29 | 0.666045046 | 0.848955091 | 0.105300771 | 1.592609412 |
|  |  | Weighted mode | 29 | 0.678857421 | 0.867875425 | 0.20402282 | 1.53172803 |
|  | APOB | Inverse variance weighted | 31 | 0.051576761 | 0.590441416 | 0.059943098 | 1.120939734 |
|  |  | Weighted median | 31 | 0.132160879 | 0.568821161 | -0.165626943 | 1.303269266 |
|  |  | Weighted mode | 31 | 0.32998285 | 0.621173784 | -0.321279188 | 1.563626757 |

**Supplementary Table 16.** The result of heterogeneity test and horizontal pleiotropic test.

| Outcomes | Drug Target | Heterogeneity test | | | | Horizontal pleiotropic test | | |
| --- | --- | --- | --- | --- | --- | --- | --- | --- |
|  |  | Method | Q | Q_df | Q_pval | egger_intercept | SE | p-value |
| PH | LDLR | MR Egger | 32.2735 | 44 | 0.9048224 | -7.89E-03 | 0.007022588 | 0.2676068 |
|  |  | Inverse variance weighted | 33.5342 | 45 | 0.8957869 |  |  |  |
|  | HMGCR | MR Egger | 12.37561 | 17 | 0.7768783 | 0.01998367 | 0.01943554 | 0.3182629 |
|  |  | Inverse variance weighted | 13.43281 | 18 | 0.7652465 |  |  |  |
|  | NPC1L1 | MR Egger | 2.463624 | 4 | 0.6511611 | -0.03271415 | 0.0253408 | 0.2662732 |
|  |  | Inverse variance weighted | 4.130221 | 5 | 0.5308239 |  |  |  |
|  | PCSK9 | MR Egger | 16.58692 | 27 | 0.9408195 | -0.000249869 | 0.006595853 | 0.9700599 |
|  |  | Inverse variance weighted | 16.58836 | 28 | 0.9562831 |  |  |  |
|  | APOB | MR Egger | 23.50014 | 29 | 0.753174 | -0.01012659 | 0.01189597 | 0.4015985 |
|  |  | Inverse variance weighted | 24.22478 | 30 | 0.7617605 |  |  |  |
|  | APOC3 | MR Egger | 52.82692 | 55 | 0.5581019 | 0.01056704 | 0.006109763 | 0.08932292 |
|  |  | Inverse variance weighted | 55.8182 | 56 | 0.4817079 |  |  |  |
|  | LPL | MR Egger | 52.82692 | 55 | 0.5581019 | 0.01056704 | 0.006109763 | 0.08932292 |
|  |  | Inverse variance weighted | 55.8182 | 56 | 0.4817079 |  |  |  |
| PI | LDLR | MR Egger | 38.88119 | 44 | 0.6902422 | -0.02746937 | 0.01489648 | 0.07192067 |
|  |  | Inverse variance weighted | 42.28159 | 45 | 0.5877741 |  |  |  |
|  | HMGCR | MR Egger | 18.35586 | 17 | 0.3667306 | 0.03227709 | 0.04285335 | 0.4616349 |
|  |  | Inverse variance weighted | 18.96842 | 18 | 0.3937707 |  |  |  |
|  | NPC1L1 | MR Egger | 1.762824 | 4 | 0.7792764 | -0.04194461 | 0.0538071 | 0.4792104 |
|  |  | Inverse variance weighted | 2.370502 | 5 | 0.7958587 |  |  |  |
|  | APOB | MR Egger | 15.97641 | 29 | 0.9758013 | -0.00502829 | 0.02523548 | 0.843454 |
|  |  | Inverse variance weighted | 16.01612 | 30 | 0.9826062 |  |  |  |
|  | APOC3 | MR Egger | 60.61526 | 55 | 0.2805245 | 0.007051159 | 0.01362369 | 0.6068367 |
|  |  | Inverse variance weighted | 60.91049 | 56 | 0.3037344 |  |  |  |
|  | LPL | MR Egger | 60.61526 | 55 | 0.2805245 | 0.007051159 | 0.01362369 | 0.6068367 |
|  |  | Inverse variance weighted | 60.91049 | 56 | 0.3037344 |  |  |  |
| Pca | LDLR | MR Egger | 31.57645 | 46 | 0.9480633 | 0.009583413 | 0.005484478 | 0.08724658 |
|  |  | Inverse variance weighted | 34.62975 | 47 | 0.9096316 |  |  |  |
|  | HMGCR | MR Egger | 9.455531 | 17 | 0.9248786 | 0.0162538 | 0.01595874 | 0.3227222 |
|  |  | Inverse variance weighted | 10.492851 | 18 | 0.9146268 |  |  |  |
|  | NPC1L1 | MR Egger | 5.498753 | 4 | 0.2398391 | -0.01690861 | 0.02697818 | 0.5648162 |
|  |  | Inverse variance weighted | 6.038754 | 5 | 0.3024662 |  |  |  |
|  | PCSK9 | MR Egger | 42.14417 | 31 | 0.08742309 | -0.005611981 | 0.006439761 | 0.3902035 |
|  |  | Inverse variance weighted | 43.17662 | 32 | 0.08972354 |  |  |  |
|  | APOB | MR Egger | 33.519 | 29 | 0.2573027 | 0.02028238 | 0.01063663 | 0.06649202 |
|  |  | Inverse variance weighted | 37.72164 | 30 | 0.157028 |  |  |  |
| AS | LDLR | MR Egger | 27.98158 | 44 | 0.9713311 | -1.02E-02 | 0.02109539 | 0.6311418 |
|  |  | Inverse variance weighted | 28.21535 | 45 | 0.9762452 |  |  |  |
|  | HMGCR | MR Egger | 9.135424 | 17 | 0.9359171 | 0.003135583 | 0.05826549 | 0.9577096 |
|  |  | Inverse variance weighted | 9.138321 | 18 | 0.9564518 |  |  |  |
|  | NPC1L1 | MR Egger | 4.963834 | 4 | 0.2910285 | -0.09189675 | 0.08480601 | 0.3394871 |
|  |  | Inverse variance weighted | 6.420984 | 5 | 0.2673823 |  |  |  |
|  | PCSK9 | MR Egger | 28.15326 | 27 | 0.4031042 | -0.01199106 | 0.02033118 | 0.5602373 |
|  |  | Inverse variance weighted | 28.51597 | 28 | 0.4373741 |  |  |  |
|  | APOB | MR Egger | 25.22752 | 29 | 0.666389 | 0.03590409 | 0.03564617 | 0.3221524 |
|  |  | Inverse variance weighted | 26.24205 | 30 | 0.6627215 |  |  |  |
|  | APOC3 | MR Egger | 43.65312 | 55 | 0.8647643 | -0.006470346 | 0.01832652 | 0.7253936 |
|  |  | Inverse variance weighted | 43.77777 | 56 | 0.8824423 |  |  |  |
|  | LPL | MR Egger | 43.65312 | 55 | 0.8647643 | -0.006470346 | 0.01832652 | 0.7253936 |
|  |  | Inverse variance weighted | 43.77777 | 56 | 0.8824423 |  |  |  |
| MI | LDLR | MR Egger | 50.10063 | 44 | 0.2442014 | 0.01412186 | 0.02580269 | 0.5869368 |
|  |  | Inverse variance weighted | 50.4417 | 45 | 0.2670838 |  |  |  |
|  | HMGCR | MR Egger | 16.5566 | 17 | 0.4847756 | -0.06833207 | 0.06693779 | 0.3216438 |
|  |  | Inverse variance weighted | 17.59869 | 18 | 0.4823687 |  |  |  |
|  | NPC1L1 | MR Egger | 4.590725 | 4 | 0.3319249 | -0.07731748 | 0.09343032 | 0.4544434 |
|  |  | Inverse variance weighted | 5.376686 | 5 | 0.3716572 |  |  |  |
|  | PCSK9 | MR Egger | 23.65533 | 27 | 0.6493586 | -0.01505517 | 0.0226595 | 0.5120634 |
|  |  | Inverse variance weighted | 24.09677 | 28 | 0.6764175 |  |  |  |
|  | APOB | MR Egger | 24.12381 | 29 | 0.7228033 | 0.02714176 | 0.04094018 | 0.5125907 |
|  |  | Inverse variance weighted | 24.56333 | 30 | 0.7459626 |  |  |  |

**Supplementary Table 17.** The results of steiger analysis.

| Outcome | Exposure | snp_r2.exposure | snp_r2.outcome | correct_causal_direction | steiger_pval |
| --- | --- | --- | --- | --- | --- |
| TT | LDLR | 0.02131312 | 0.000231809 | TRUE | 0 |
|  | HMGCR | 0.006021365 | 0.000440789 | TRUE | 3.21E-98 |
|  | LPL | 0.04028851 | 0.000525527 | TRUE | 0 |
| BT | HMGCR | 0.006021365 | 0.000322061 | TRUE | 4.83E-103 |
| SHBG | LDLR | 0.02131312 | 0.000196475 | TRUE | 0 |
|  | HMGCR | 0.006021365 | 0.000105116 | TRUE | 3.17E-131 |
|  | LPL | 0.04028851 | 0.000652732 | TRUE | 0 |
|  | PCSK9 | 0.01044584 | 0.000239005 | TRUE | 3.84E-217 |
|  | APOC3 | 0.03749518 | 0.000261374 | TRUE | 0 |
| PH | PCSK10 | 0.008987007 | 0.000280343 | TRUE | 5.86E-98 |
|  | LDLR | 0.01932364 | 0.000545444 | TRUE | 2.08E-214 |
| PCa | NPC1L1 | 0.001016655 | 6.49E-05 | TRUE | 2.12E-19 |
|  | HMGCR | 0.006021365 | 0.000140017 | TRUE | 6.09E-137 |
| AS | LDLR | 0.01932364 | 1.81E-05 | TRUE | 0 |
| MI | HMGCR | 0.006021365 | 0.00046472 | TRUE | 3.73E-45 |

**Supplementary Table 18.** The results of Mediation Analysis.

| Drug targets | Mediation | Outcome | β1 | SE1 | β2 | SE2 | b | S | Z | P-vale |
| --- | --- | --- | --- | --- | --- | --- | --- | --- | --- | --- |
| PCSK9 | SHBG | PH | -0.092 | 0.020 | 0.024 | 0.037 | -0.002 | 0.002 | -1.073 | 0.283 |
| LDLR | TT | PH | -0.047 | 0.015 | 0.026 | 0.043 | -0.001 | 0.001 | -0.919 | 0.358 |
|  | SHBG |  | -0.046 | 0.015 | 0.024 | 0.037 | -0.001 | 0.001 | -0.974 | 0.330 |
| NPC1L1 | BT | PCa | -0.148 | 0.074 | 0.077 | 0.068 | -0.011 | 0.012 | -0.943 | 0.346 |
| HMGCR | TT | PCa | 0.235 | 0.031 | -0.006 | 0.048 | -0.001 | 0.007 | -0.195 | 0.845 |
|  | BT |  | 0.194 | 0.031 | 0.077 | 0.068 | 0.015 | 0.008 | 1.892 | 0.059 |
|  | SHBG |  | 0.094 | 0.030 | -0.077 | 0.042 | -0.007 | 0.004 | -1.685 | 0.092 |
| LDLR | TT | AS | -0.047 | 0.015 | 0.080 | 0.131 | -0.004 | 0.011 | -0.357 | 0.721 |
|  | SHBG |  | -0.046 | 0.015 | -0.015 | 0.110 | 0.001 | 0.002 | 0.384 | 0.701 |
| HMGCR | TT | MI | 0.235 | 0.031 | 0.170 | 0.150 | 0.040 | 0.027 | 1.507 | 0.132 |
|  | BT |  | 0.194 | 0.031 | -0.117 | 0.226 | -0.023 | 0.027 | -0.839 | 0.402 |
|  | SHBG |  | 0.094 | 0.030 | 0.107 | 0.122 | 0.010 | 0.013 | 0.755 | 0.450 |
