# Supplementary material for: Genetic association of lipid-lowering drug target genes with erectile dysfunction and male reproductive health
Source: Front Endocrinol (Lausanne). 2024 Feb 8;15:1362499. doi: 10.3389/fendo.2024.1362499 (PMC10881712; doi:10.3389/fendo.2024.1362499)

Supplementary figure 1. Leave-one-out analysis of genetically proxied inhibitors / agonists on ED(FinnGen).

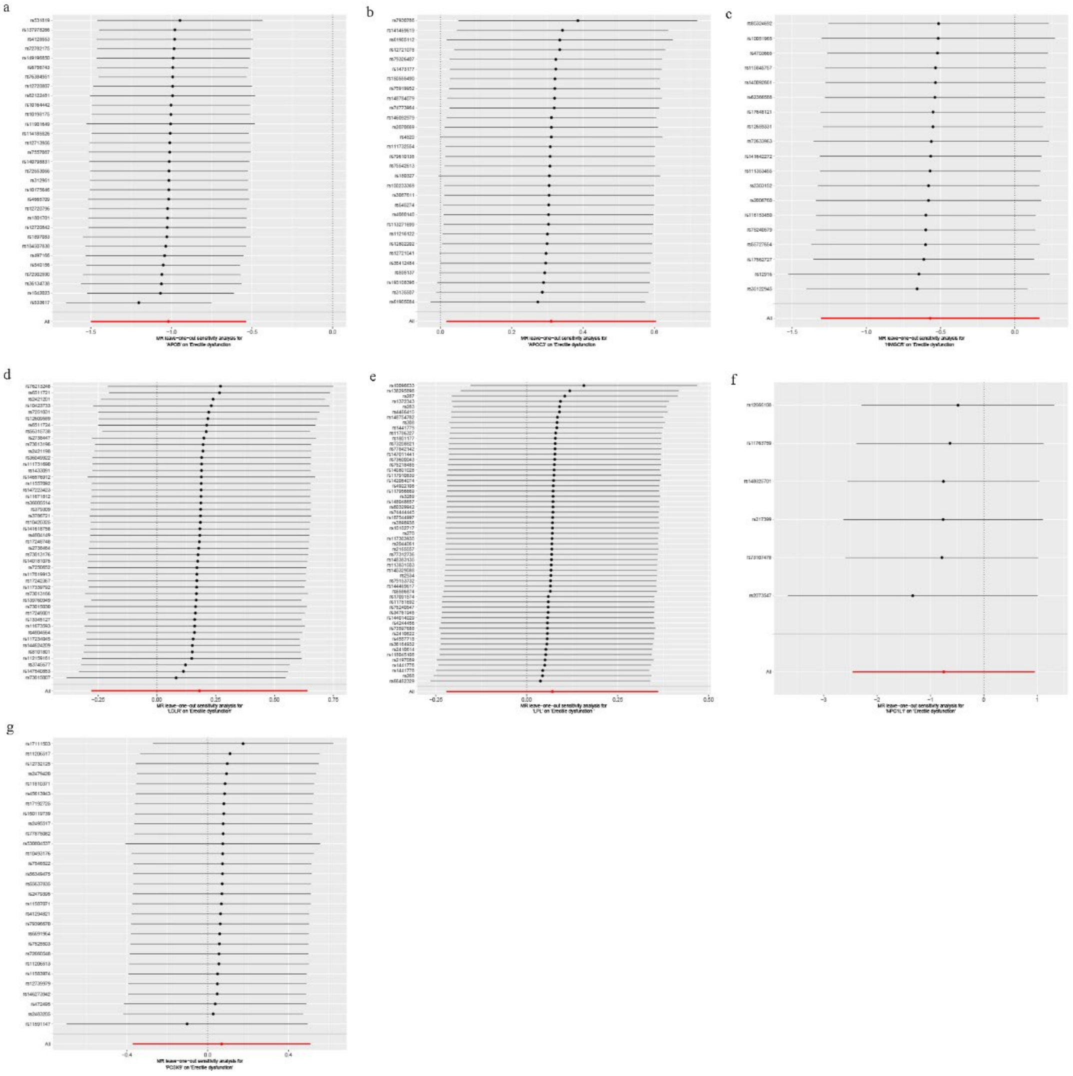

Supplementary figure 2. Leave-one-out analysis of genetically proxied inhibitors / agonists on ED(ebi).

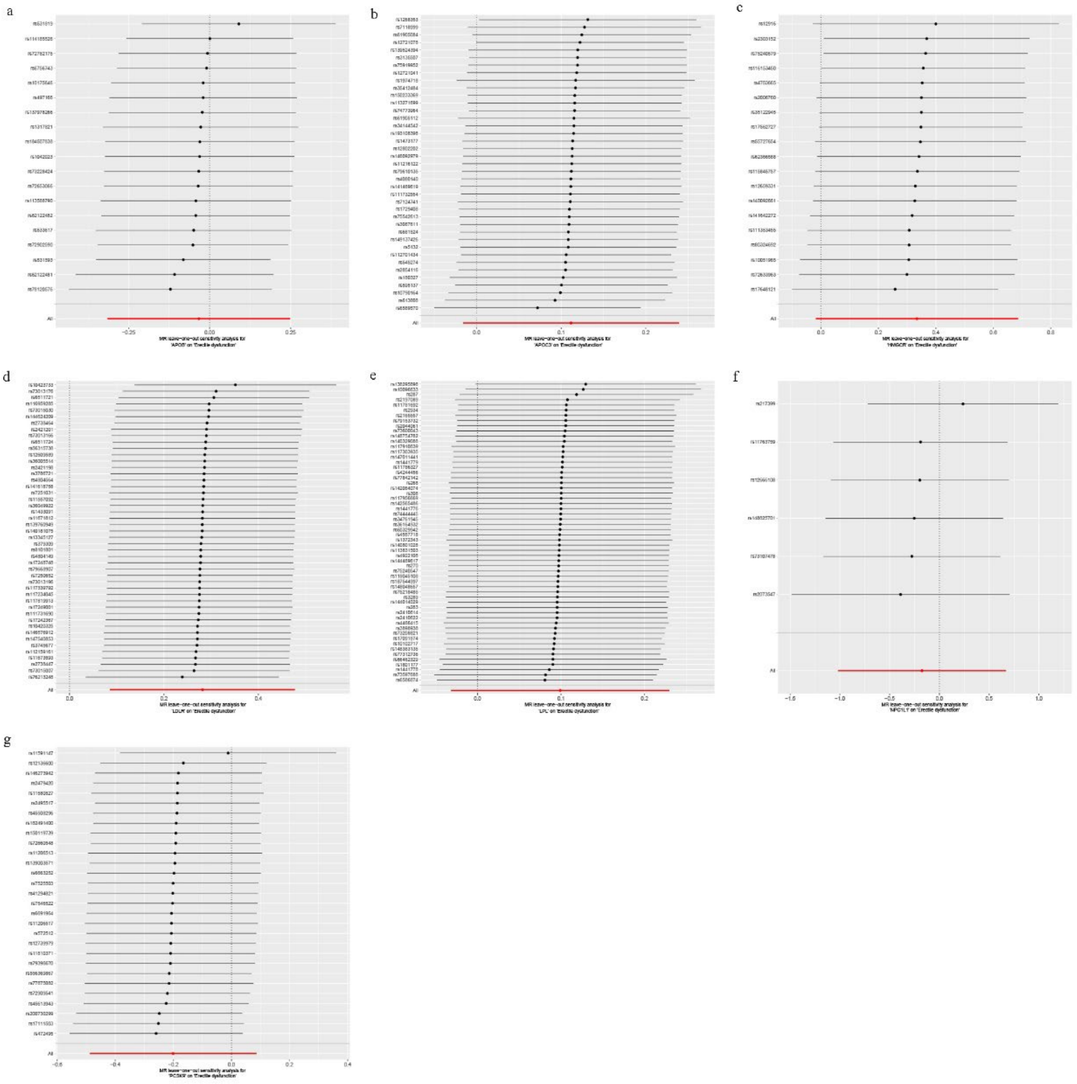

Supplementary figure 3. Leave-one-out analysis of genetically proxied inhibitors / agonists on TT.

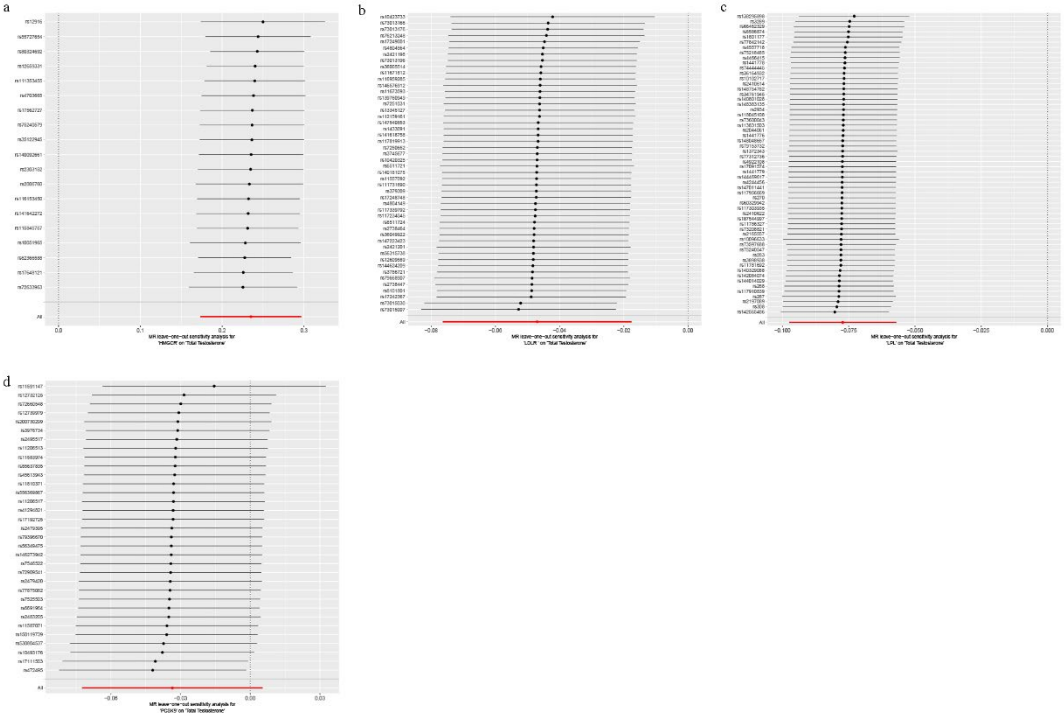

Supplementary figure 4. Leave-one-out analysis of genetically proxied inhibitors / agonists on BT.

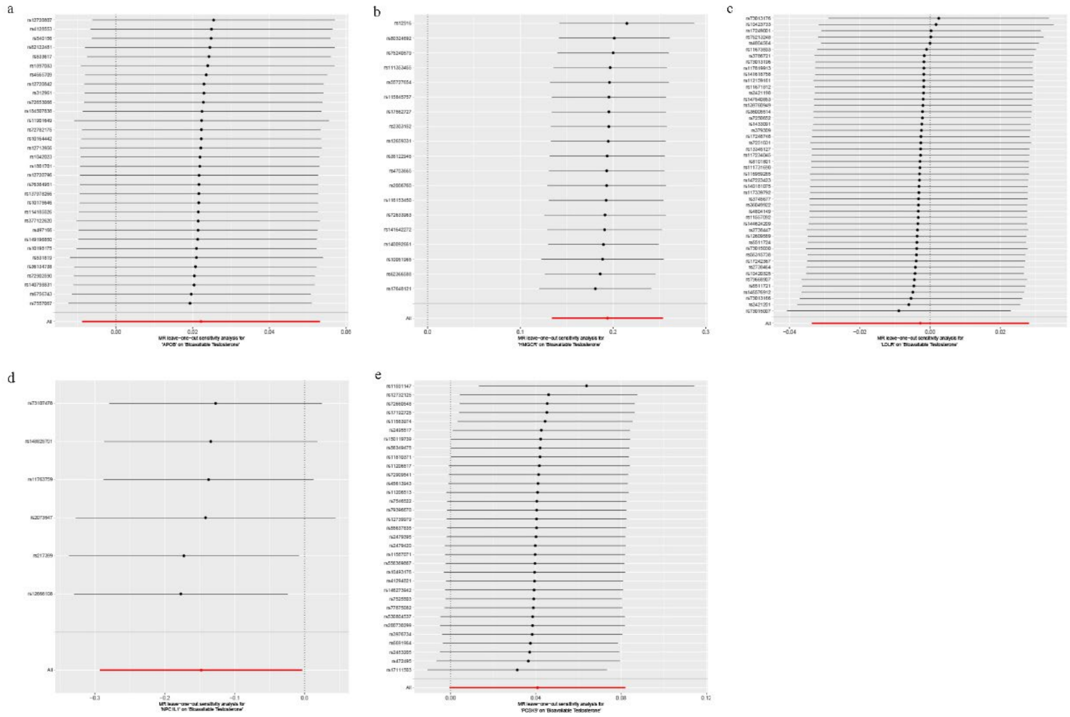

Supplementary figure 5. Leave-one-out analysis of genetically proxied inhibitors / agonists on E2.

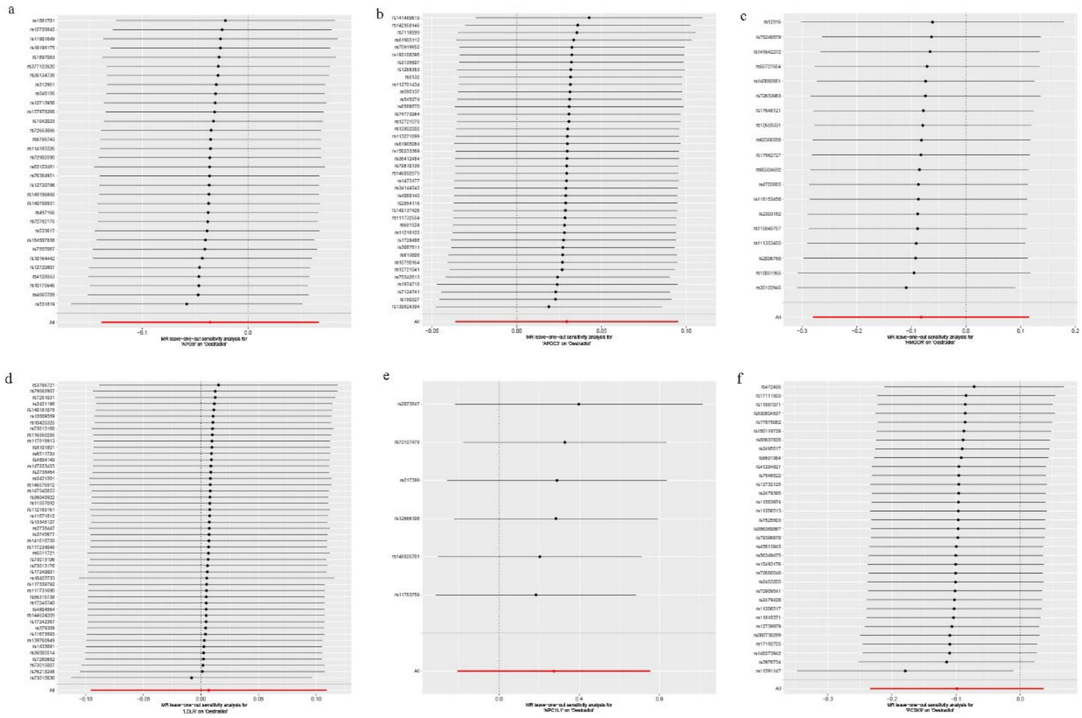

Supplementary figure 6. Leave-one-out analysis of genetically proxied inhibitors / agonists on SHBG.

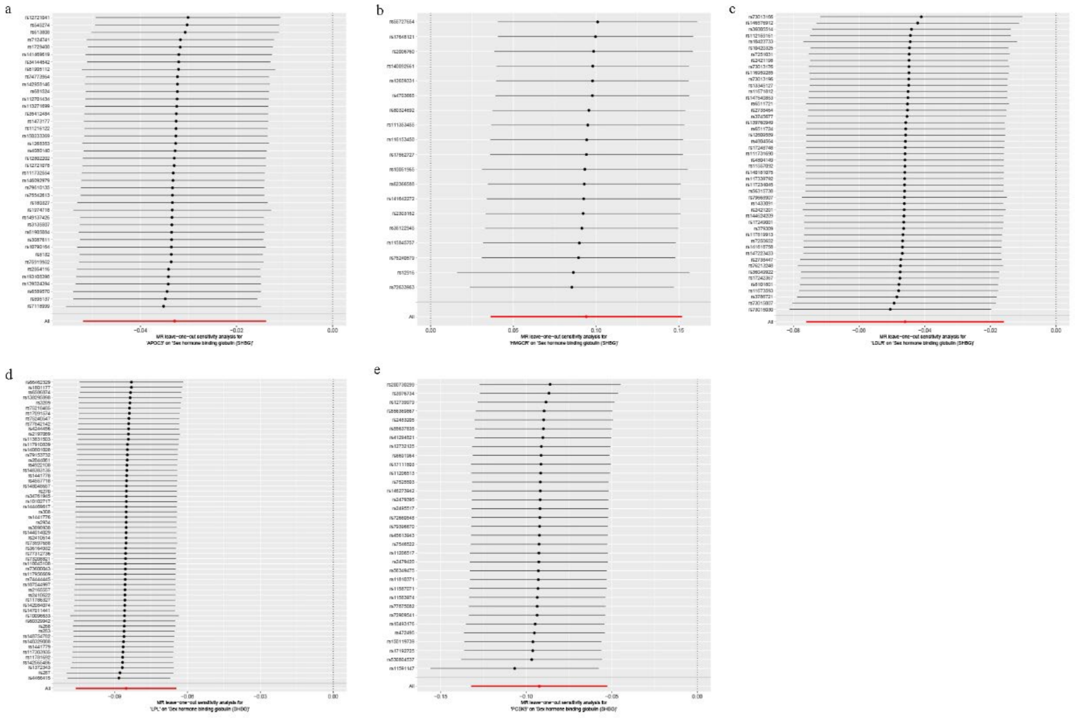

Supplementary figure 7. Leave-one-out analysis of genetically proxied inhibitors / agonists on PH.

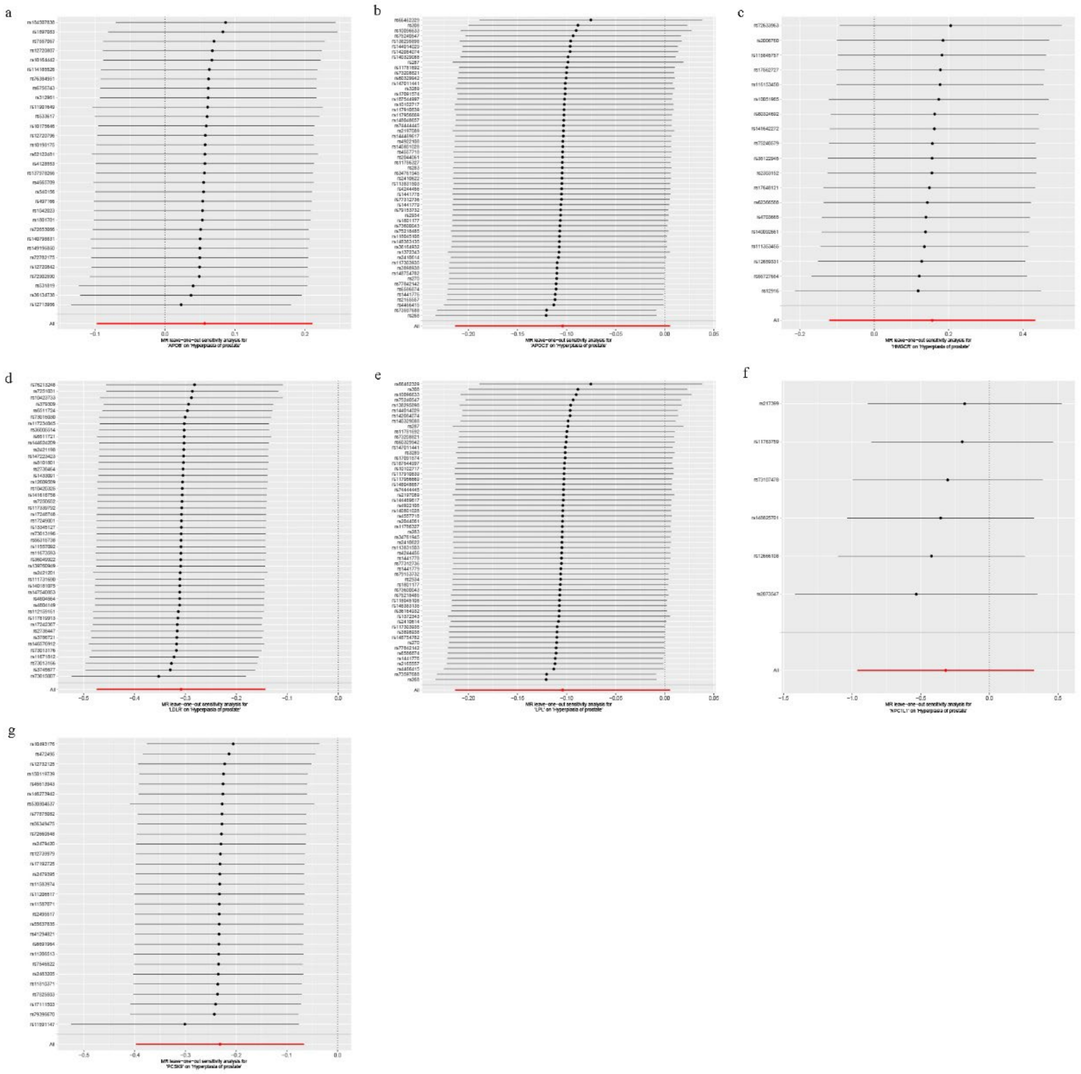

Supplementary figure 8. Leave-one-out analysis of genetically proxied inhibitors / agonists on PI.

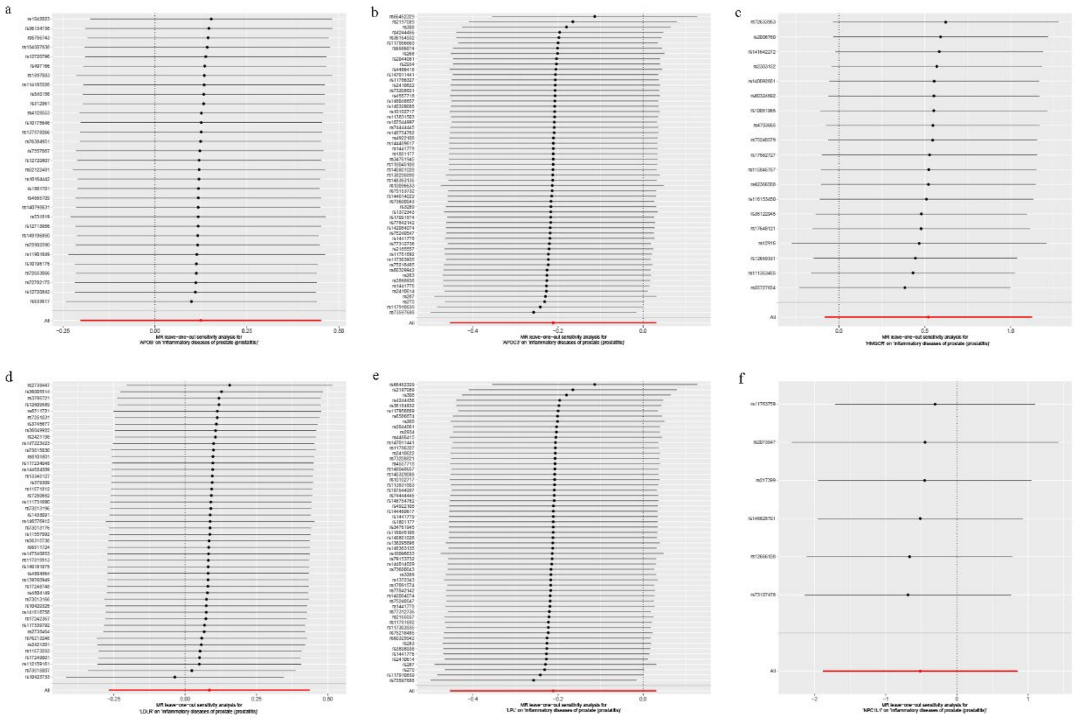

Supplementary figure 9. Leave-one-out analysis of genetically proxied inhibitors / agonists on PCa.

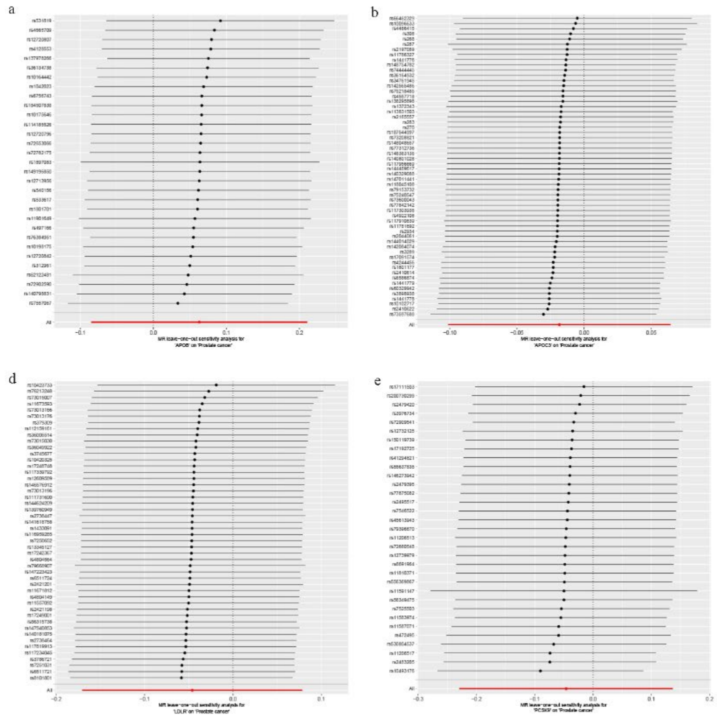

Supplementary figure 10. Leave-one-out analysis of genetically proxied inhibitors / agonists on AS.

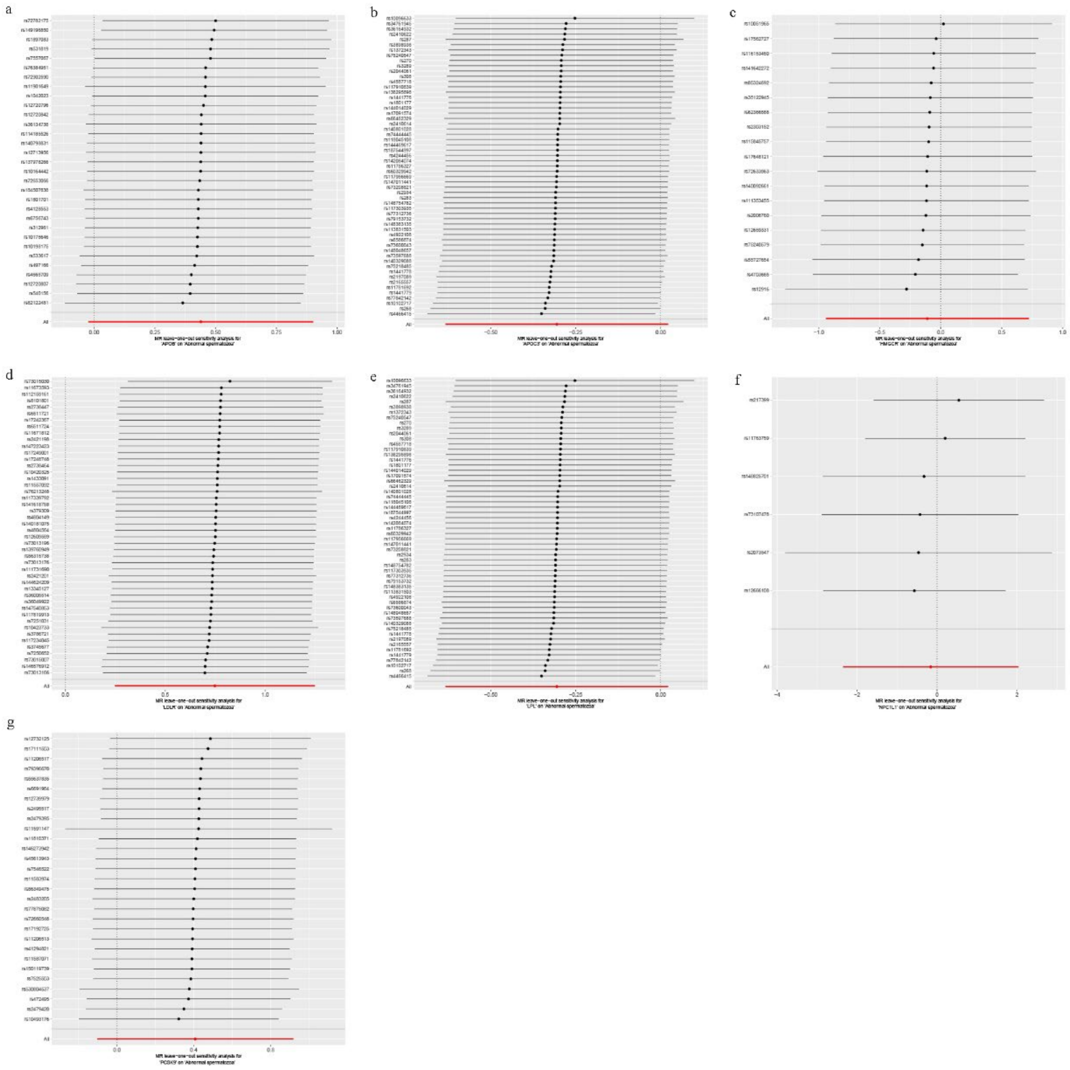

Supplementary figure 11. Leave-one-out analysis of genetically proxied inhibitors / agonists on MI.

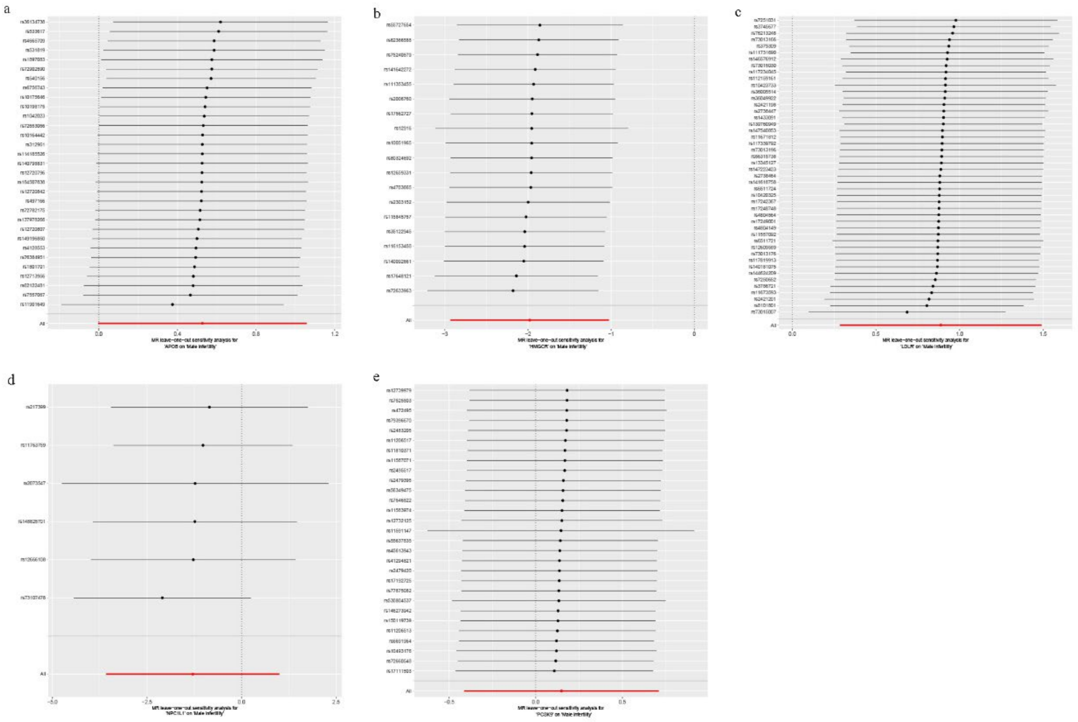

Supplementary figure 12. MR analysis of association between drug targets and sex hormones (TT and BT).

Outcome	Target	method	nsnp	pval	OR (95% CI)
TT	LDLR	Inverse variance weighted	48	0.0017	1.0481 (1.0187 - 1.0775)
		Weighted median	48	0.0025	1.0669 (1.0250 - 1.1089)
		Weighted mode	48	0.0222	1.0633 (1.0124 - 1.1142)
	HMGCR	Inverse variance weighted	19	<0.001	0.7904 (0.7288 - 0.8521)
		Weighted median	19	<0.001	0.8104 (0.7318 - 0.8891)
		Weighted mode	19	<0.001	0.7968 (0.7214 - 0.8722)
	PCSK9	Inverse variance weighted	33	0.0912	1.0341 (0.9952 - 1.0730)
		Weighted median	33	0.0229	1.0688 (1.0115 - 1.1262)
		Weighted mode	33	0.0872	1.0589 (0.9953 - 1.1225)
	LPL	Inverse variance weighted	58	<0.001	1.0803 (1.0601 - 1.1006)
		Weighted median	58	<0.001	1.0762 (1.0457 - 1.1067)
		Weighted mode	58	<0.001	1.0754 (1.0445 - 1.1063)
BT	LDLR	Inverse variance weighted	48	0.8606	1.0028 (0.9719 - 1.0337)
		Weighted median	48	0.4895	0.9844 (0.9397 - 1.0291)
		Weighted mode	48	0.7138	0.9905 (0.9396 - 1.0413)
	HMGCR	Inverse variance weighted	19	<0.001	0.8237 (0.7638 - 0.8836)
		Weighted median	19	<0.001	0.8436 (0.7636 - 0.9236)
		Weighted mode	19	<0.001	0.8342 (0.7537 - 0.9147)
	NPC1L1	Inverse variance weighted	6	0.0448	1.1595 (1.0150 - 1.3041)
		Weighted median	6	0.1008	1.1587 (0.9828 - 1.3347)
		Weighted mode	6	0.2435	1.1613 (0.9396 - 1.3831)
	PCSK9	Inverse variance weighted	6	0.0448	1.1595 (1.0150 - 1.3041)
		Weighted median	6	0.1008	1.1587 (0.9828 - 1.3347)
		Weighted mode	6	0.2435	1.1613 (0.9396 - 1.3831)
	APOB	Inverse variance weighted	32	0.1609	0.9781 (0.9471 - 1.0091)
		Weighted median	32	0.3349	0.9793 (0.9368 - 1.0218)
		Weighted mode	32	0.4154	0.9810 (0.9356 - 1.0265)

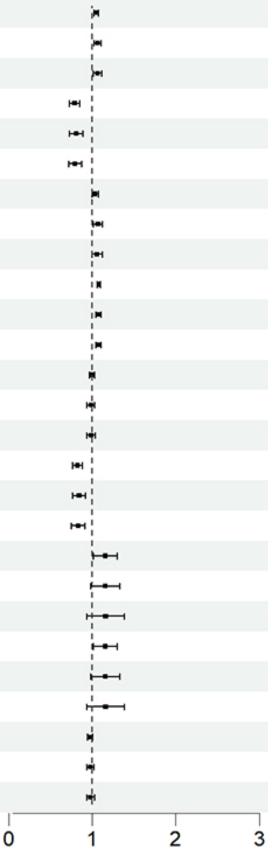

Supplementary figure 13. MR analysis of association between drug targets and sex hormones (E2 and SHBG).

Outcome	Target	method	nsnp	pval	OR (95% CI)
E2	LDLR	Inverse variance weighted	48	0.8978	0.9933 (0.8909 − 1.0957)
		Weighted median	48	0.9051	0.9912 (0.8466 − 1.1359)
		Weighted mode	48	0.9992	1.0001 (0.8450 − 1.1552)
	HMGCR	Inverse variance weighted	19	0.4158	1.0856 (0.8877 − 1.2835)
		Weighted median	19	0.3485	1.1339 (0.8712 − 1.3967)
		Weighted mode	19	0.4645	1.1107 (0.8354 − 1.3860)
	NPC1L1	Inverse variance weighted	6	0.2652	0.7607 (0.2795 − 1.2418)
		Weighted median	6	0.5664	0.8472 (0.2805 − 1.4140)
		Weighted mode	6	0.7309	0.8824 (0.2084 − 1.5564)
	PCSK9	Inverse variance weighted	33	0.1522	1.1038 (0.9686 − 1.2390)
		Weighted median	33	0.7671	0.9708 (0.7744 − 1.1671)
		Weighted mode	33	0.9946	0.9993 (0.7986 − 1.2000)
	APOB	Inverse variance weighted	32	0.4942	1.0365 (0.9338 − 1.1392)
		Weighted median	32	0.5779	1.0429 (0.8949 − 1.1910)
		Weighted mode	32	0.5284	1.0496 (0.9009 − 1.1983)
	APOC3	Inverse variance weighted	40	0.3791	0.9709 (0.9051 − 1.0367)
		Weighted median	40	0.4539	0.9653 (0.8728 − 1.0578)
		Weighted mode	40	0.3712	0.9597 (0.8705 − 1.0488)
SHBG	LDLR	Inverse variance weighted	48	0.0027	1.0470 (1.0170 − 1.0771)
		Weighted median	48	0.0304	1.0493 (1.0057 − 1.0929)
		Weighted mode	48	0.0632	1.0492 (0.9997 − 1.0986)
	HMGCR	Inverse variance weighted	19	0.0014	0.9102 (0.8524 − 0.9680)
		Weighted median	19	0.0079	0.8993 (0.8210 − 0.9776)
		Weighted mode	19	0.0165	0.8962 (0.8150 − 0.9775)
	PCSK9	Inverse variance weighted	33	<0.001	1.0968 (1.0570 − 1.1366)
		Weighted median	33	0.0278	1.0691 (1.0096 − 1.1286)
		Weighted mode	33	0.0274	1.0729 (1.0132 − 1.1326)
	APOC3	Inverse variance weighted	40	<0.001	1.0334 (1.0144 − 1.0525)
		Weighted median	40	0.0252	1.0296 (1.0041 − 1.0552)
		Weighted mode	40	0.0183	1.0310 (1.0067 − 1.0552)
	LPL	Inverse variance weighted	58	<0.001	1.0890 (1.0683 − 1.1097)
		Weighted median	58	<0.001	1.0873 (1.0563 − 1.1183)
		Weighted mode	58	<0.001	1.0868 (1.0551 − 1.1185)

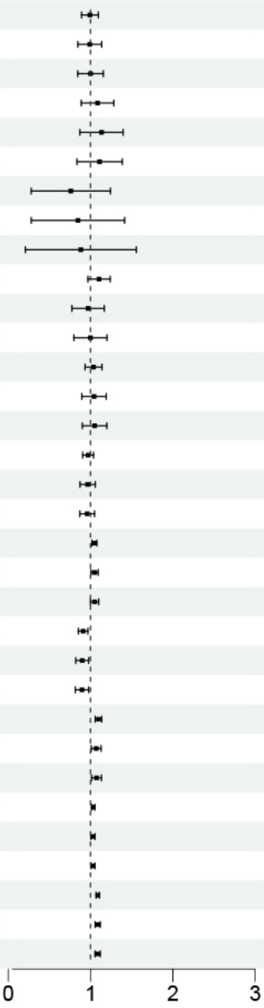

Supplementary figure 14. MR analysis of association between drug targets and male diseases (PH, PI and PCa).

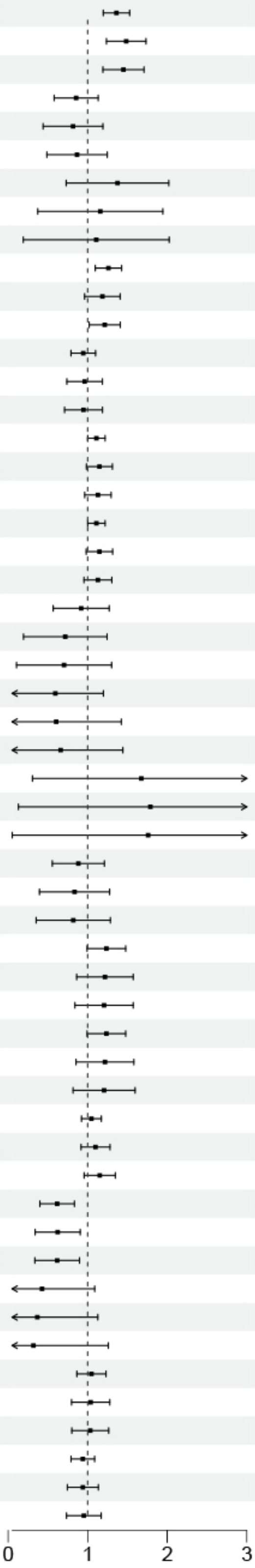

Outcome	Target	method	nsnp	pval	OR (95% CI)
PH	LDLR	Inverse variance weighted	46	<0.001	1.3616 (1.1960 − 1.5273)
		Weighted median	46	0.0018	1.4844 (1.2361 − 1.7327)
		Weighted mode	46	0.0071	1.4498 (1.1915 − 1.7081)
	HMGCR	Inverse variance weighted	19	0.2690	0.8554 (0.5785 − 1.1323)
		Weighted median	19	0.2880	0.8158 (0.4400 − 1.1916)
		Weighted mode	19	0.4690	0.8665 (0.4872 − 1.2459)
	NPC1L1	Inverse variance weighted	6	0.3334	1.3746 (0.7299 − 2.0193)
		Weighted median	6	0.7129	1.1590 (0.3732 − 1.9448)
		Weighted mode	6	0.8356	1.1077 (0.1910 − 2.0243)
	PCSK9	Inverse variance weighted	29	0.0061	1.2610 (1.0952 − 1.4268)
		Weighted median	29	0.1368	1.1850 (0.9614 − 1.4087)
		Weighted mode	29	0.0612	1.2144 (1.0192 − 1.4097)
	APOB	Inverse variance weighted	31	0.4706	0.9448 (0.7906 − 1.0990)
		Weighted median	31	0.7347	0.9621 (0.7388 − 1.1855)
		Weighted mode	31	0.6616	0.9478 (0.7099 − 1.1856)
	APOC3	Inverse variance weighted	57	0.0623	1.1097 (1.0002 − 1.2192)
		Weighted median	57	0.0972	1.1479 (0.9849 − 1.3108)
		Weighted mode	57	0.1584	1.1286 (0.9627 − 1.2945)
	LPL	Inverse variance weighted	57	0.0623	1.1097 (1.0002 − 1.2192)
		Weighted median	57	0.1030	1.1479 (0.9820 − 1.3138)
		Weighted mode	57	0.1820	1.1286 (0.9532 − 1.3040)
PI	LDLR	Inverse variance weighted	46	0.6372	0.9188 (0.5669 − 1.2707)
		Weighted median	46	0.2172	0.7186 (0.1935 − 1.2436)
		Weighted mode	46	0.2546	0.7035 (0.1060 − 1.3009)
	HMGCR	Inverse variance weighted	19	0.0909	0.5940 (−0.0099 − 1.1978)
		Weighted median	19	0.2280	0.6044 (−0.2142 − 1.4230)
		Weighted mode	19	0.3120	0.6604 (−0.1215 − 1.4424)
	NPC1L1	Inverse variance weighted	6	0.4603	1.6739 (0.3063 − 3.0415)
		Weighted median	6	0.4922	1.7882 (0.1297 − 3.4466)
		Weighted mode	6	0.5452	1.7601 (0.0515 − 3.4688)
	APOB	Inverse variance weighted	31	0.4556	0.8829 (0.5557 − 1.2101)
		Weighted median	31	0.4226	0.8350 (0.3944 − 1.2756)
		Weighted mode	31	0.4079	0.8190 (0.3526 − 1.2853)
	APOC3	Inverse variance weighted	57	0.0874	1.2353 (0.9930 − 1.4777)
		Weighted median	57	0.2788	1.2171 (0.8615 − 1.5726)
		Weighted mode	57	0.3200	1.2062 (0.8400 − 1.5723)
	LPL	Inverse variance weighted	57	0.0874	1.2353 (0.9930 − 1.4777)
		Weighted median	57	0.2890	1.2171 (0.8538 − 1.5804)
		Weighted mode	57	0.3500	1.2062 (0.8165 − 1.5958)
PCa	LDLR	Inverse variance weighted	48	0.4685	1.0472 (0.9225 − 1.1718)
		Weighted median	48	0.3102	1.0987 (0.9169 − 1.2806)
		Weighted mode	48	0.1652	1.1517 (0.9554 − 1.3480)
	HMGCR	Inverse variance weighted	19	<0.001	0.6162 (0.3991 − 0.8333)
		Weighted median	19	0.0011	0.6231 (0.3399 − 0.9064)
		Weighted mode	19	0.0034	0.6168 (0.3360 − 0.8977)
	NPC1L1	Inverse variance weighted	6	0.0116	0.4268 (−0.2347 − 1.0883)
		Weighted median	6	0.0098	0.3670 (−0.3934 − 1.1274)
		Weighted mode	6	0.0628	0.3184 (−0.6225 − 1.2592)
	PCSK9	Inverse variance weighted	33	0.6210	1.0473 (0.8641 − 1.2306)
		Weighted median	33	0.7637	1.0375 (0.7973 − 1.2778)
		Weighted mode	33	0.7891	1.0324 (0.8008 − 1.2640)
	APOB	Inverse variance weighted	31	0.4052	0.9391 (0.7910 − 1.0871)
		Weighted median	31	0.5311	0.9396 (0.7448 − 1.1345)
		Weighted mode	31	0.6595	0.9518 (0.7340 − 1.1695)

Supplementary figure 15. MR analysis of association between drug targets and male diseases (AS and MI).

Outcome	Target	method	nsnp	pval	OR (95% CI)
AS	LDLR	Inverse variance weighted	46	0.0034	0.4730 (−0.0283 − 0.9743)
		Weighted median	46	0.0186	0.4303 (−0.2716 − 1.1323)
		Weighted mode	46	0.0195	0.4105 (−0.3099 − 1.1309)
	HMGCR	Inverse variance weighted	19	0.7960	1.1161 (0.2838 − 1.9485)
		Weighted median	19	0.8960	0.9294 (−0.1640 − 2.0229)
		Weighted mode	19	0.8700	0.9041 (−0.2824 − 2.0907)
	NPC1L1	Inverse variance weighted	6	0.8889	1.1695 (−1.0263 − 3.3653)
		Weighted median	6	0.7682	0.6921 (−1.7553 − 3.1395)
		Weighted mode	6	0.6426	0.5040 (−2.2181 − 3.2260)
	PCSK9	Inverse variance weighted	29	0.1177	0.6652 (0.1546 − 1.1759)
		Weighted median	29	0.1813	0.6353 (−0.0299 − 1.3005)
		Weighted mode	29	0.1279	0.6082 (−0.0130 − 1.2294)
	APOB	Inverse variance weighted	31	0.0631	0.6446 (0.1816 − 1.1076)
		Weighted median	31	0.2042	0.6643 (0.0329 − 1.2957)
		Weighted mode	31	0.2907	0.7040 (0.0645 − 1.3436)
	APOC3	Inverse variance weighted	57	0.0678	1.3571 (1.0294 − 1.6848)
		Weighted median	57	0.0511	1.5951 (1.1260 − 2.0642)
		Weighted mode	57	0.0990	1.4877 (1.0236 − 1.9518)
	LPL	Inverse variance weighted	57	0.0678	1.3571 (1.0294 − 1.6848)
		Weighted median	57	0.0488	1.5951 (1.1306 − 2.0596)
		Weighted mode	57	0.0902	1.4877 (1.0361 − 1.9392)
MI	LDLR	Inverse variance weighted	46	0.0039	0.4116 (−0.1919 − 1.0151)
		Weighted median	46	0.1190	0.5145 (−0.3211 − 1.3500)
		Weighted mode	46	0.1544	0.5114 (−0.3962 − 1.4189)
	HMGCR	Inverse variance weighted	19	<0.001	7.2391 (6.2856 − 8.1926)
		Weighted median	19	0.0028	7.9172 (6.5608 − 9.2736)
		Weighted mode	19	0.0071	8.0886 (6.7384 − 9.4387)
	NPC1L1	Inverse variance weighted	6	0.2710	3.6279 (1.3333 − 5.9226)
		Weighted median	6	0.3027	4.0221 (1.3755 − 6.6687)
		Weighted mode	6	0.3709	4.4871 (1.4928 − 7.4813)
	PCSK9	Inverse variance weighted	29	0.6055	0.8623 (0.3000 − 1.4246)
		Weighted median	29	0.6660	0.8490 (0.1053 − 1.5926)
		Weighted mode	29	0.6789	0.8679 (0.2040 − 1.5317)
	APOB	Inverse variance weighted	31	0.0516	0.5904 (0.0599 − 1.1209)
		Weighted median	31	0.1322	0.5688 (−0.1656 − 1.3033)
		Weighted mode	31	0.3300	0.6212 (−0.3213 − 1.5636)

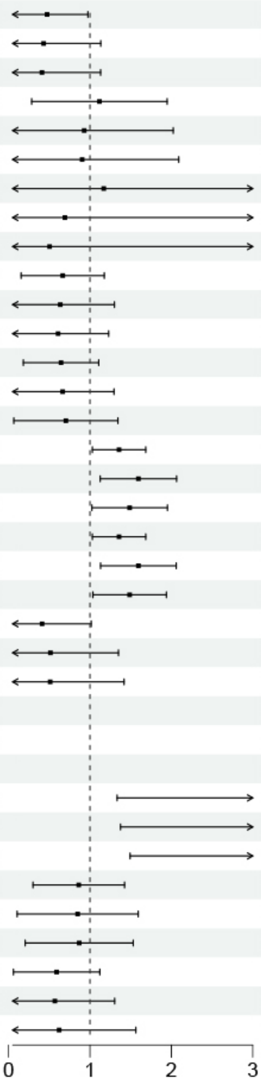

Supplement: Supplementary file 2 [file Image_1.pdf]
